# The structure of the mouse ADAT2/ADAT3 complex reveals the molecular basis for mammalian tRNA wobble adenosine-to-inosine deamination

**DOI:** 10.1093/nar/gkab436

**Published:** 2021-05-31

**Authors:** Elizabeth Ramos-Morales, Efil Bayam, Jordi Del-Pozo-Rodríguez, Thalia Salinas-Giegé, Martin Marek, Peggy Tilly, Philippe Wolff, Edouard Troesch, Eric Ennifar, Laurence Drouard, Juliette D Godin, Christophe Romier

**Affiliations:** Université de Strasbourg, CNRS, INSERM, Institut de Génétique et de Biologie Moléculaire et Cellulaire (IGBMC), UMR 7104, U 1258, 1 rue Laurent Fries, B.P. 10142, 67404, Illkirch Cedex, France; Université de Strasbourg, CNRS, INSERM, Institut de Génétique et de Biologie Moléculaire et Cellulaire (IGBMC), UMR 7104, U 1258, 1 rue Laurent Fries, B.P. 10142, 67404, Illkirch Cedex, France; Université de Strasbourg, CNRS, INSERM, Institut de Génétique et de Biologie Moléculaire et Cellulaire (IGBMC), UMR 7104, U 1258, 1 rue Laurent Fries, B.P. 10142, 67404, Illkirch Cedex, France; Institut de biologie moléculaire des plantes-CNRS, Université de Strasbourg, 12 rue du Général Zimmer, 67084 Strasbourg, France; Université de Strasbourg, CNRS, INSERM, Institut de Génétique et de Biologie Moléculaire et Cellulaire (IGBMC), UMR 7104, U 1258, 1 rue Laurent Fries, B.P. 10142, 67404, Illkirch Cedex, France; Université de Strasbourg, CNRS, INSERM, Institut de Génétique et de Biologie Moléculaire et Cellulaire (IGBMC), UMR 7104, U 1258, 1 rue Laurent Fries, B.P. 10142, 67404, Illkirch Cedex, France; Université de Strasbourg, CNRS, Architecture et Réactivité de l’ARN, UPR 9002, 67000 Strasbourg, France; Université de Strasbourg, CNRS, INSERM, Institut de Génétique et de Biologie Moléculaire et Cellulaire (IGBMC), UMR 7104, U 1258, 1 rue Laurent Fries, B.P. 10142, 67404, Illkirch Cedex, France; Université de Strasbourg, CNRS, Architecture et Réactivité de l’ARN, UPR 9002, 67000 Strasbourg, France; Institut de biologie moléculaire des plantes-CNRS, Université de Strasbourg, 12 rue du Général Zimmer, 67084 Strasbourg, France; Université de Strasbourg, CNRS, INSERM, Institut de Génétique et de Biologie Moléculaire et Cellulaire (IGBMC), UMR 7104, U 1258, 1 rue Laurent Fries, B.P. 10142, 67404, Illkirch Cedex, France; Université de Strasbourg, CNRS, INSERM, Institut de Génétique et de Biologie Moléculaire et Cellulaire (IGBMC), UMR 7104, U 1258, 1 rue Laurent Fries, B.P. 10142, 67404, Illkirch Cedex, France

## Abstract

Post-transcriptional modification of tRNA wobble adenosine into inosine is crucial for decoding multiple mRNA codons by a single tRNA. The eukaryotic wobble adenosine-to-inosine modification is catalysed by the ADAT (ADAT2/ADAT3) complex that modifies up to eight tRNAs, requiring a full tRNA for activity. Yet, ADAT catalytic mechanism and its implication in neurodevelopmental disorders remain poorly understood. Here, we have characterized mouse ADAT and provide the molecular basis for tRNAs deamination by ADAT2 as well as ADAT3 inactivation by loss of catalytic and tRNA-binding determinants. We show that tRNA binding and deamination can vary depending on the cognate tRNA but absolutely rely on the eukaryote-specific ADAT3 N-terminal domain. This domain can rotate with respect to the ADAT catalytic domain to present and position the tRNA anticodon-stem-loop correctly in ADAT2 active site. A founder mutation in the ADAT3 N-terminal domain, which causes intellectual disability, does not affect tRNA binding despite the structural changes it induces but most likely hinders optimal presentation of the tRNA anticodon-stem-loop to ADAT2.

## Graphical Abstract

**Graphical abstract:**
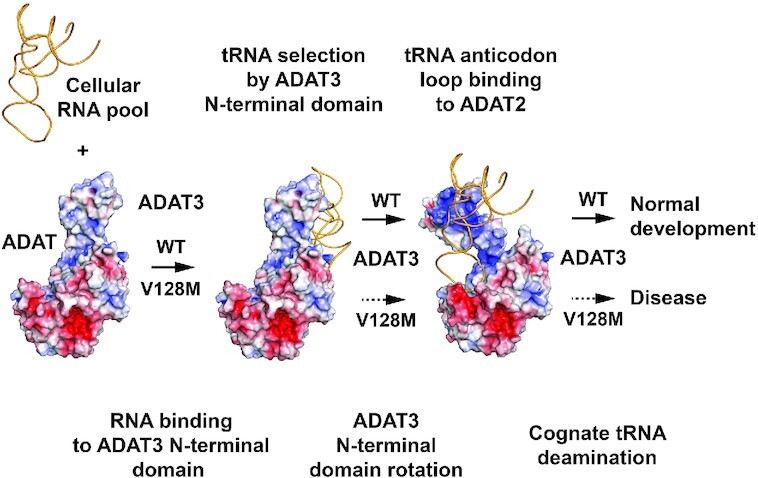
ADAT catalyses tRNAs wobble adenosine-to-inosine modification through ADAT3 recognition of tRNAs 3D structure and anticodon-loop presentation to ADAT2 catalytic subunit. ADAT3 V128M mutation hampers presentation, leading to neurodevelopmental disorders.

## INTRODUCTION

All ribonucleic acid molecules undergo a large number of post-transcriptional covalent modifications (1). Transfer RNAs are among the most modified RNA species, where modifications play a role in tRNA folding, stabilization and decoding, but can also provide a checkpoint for the interaction between translation and other cellular processes and environmental cues (2–5). Mutations in genes coding for tRNA-modifying enzymes cause numerous diseases. Intriguingly, the vast majority of genetic disorders linked to these mutations are neurological disorders, particularly neurodevelopmental disorders (NDDs), highlighting the importance of tRNA editing for proper brain development (6–8). How dysregulation of tRNA modifications causes these diseases remains poorly understood.

Among the tRNA modifications, a large set is located in the anticodon-stem-loop (ASL) of tRNAs, playing notably a major role in decoding. This is particularly the case for the modifications of nucleoside 34 of the ASL, which faces the third base of mRNA codons during translation. Modification of adenosine into inosine (A-to-I) at position 34 led Crick to propose the wobble hypothesis, thereby providing an explanation to the degeneracy of the genetic code, inosine being able to pair with uridine, adenosine and cytosine in contrast to the typical Watson–Crick base pairing made by adenosine with uridine (9). Since this initial hypothesis, the number of modifications discovered for the wobble base at position 34 and in other nucleosides of the ASL has increased, enabling a better understanding of codon decoding by tRNAs (10).

Wobble A-to-I modification is conserved among prokaryotes and eukaryotes (10,11). Yet, whereas only tRNA^Arg^(ACG) is modified in prokaryotes, up to eight tRNAs are inosine-modified in eukaryotes (Ala(AGC), Arg(ACG), Ile(AAU), Leu(AAG), Pro(AGG), Ser(AGA), Thr(AGU) and Val(AAC); Supplementary Table S1) (1). Wobble A-to-I modification occurs through a deamination reaction catalysed by enzymes that belong to the larger cytidine deaminase (CDA) family (12).

In prokaryotes, the essential homodimeric TadA enzyme is responsible for this reaction that only requires the ASL of the cognate tRNA^Arg^(ACG) for activity (13). The structures of various prokaryotic TadA enzymes have been solved, showing that the fold and the coordination of the catalytic zinc ion through one histidine, two cysteines and a water molecule are conserved within the cytidine deaminase family, also including the catalytic glutamate proposed to shuttle a proton from the aforementioned water to the wobble adenine (14–16).

The structure of *Staphylococcus aureus* TadA in complex with an ASL containing Nebularine, a non-hydrolysable adenosine analogue, has further shed light on the recognition of prokaryotic tRNA^Arg^(ACG) by TadA (17). Specifically, the ASL undergoes significant conformational changes upon binding to TadA, and five bases of the anticodon stem-loop are splayed and recognized within different pockets. Bases 32–38 of the ASL are sufficient for specific recognition of tRNA^Arg^(ACG) by TadA.

**Figure 1.**
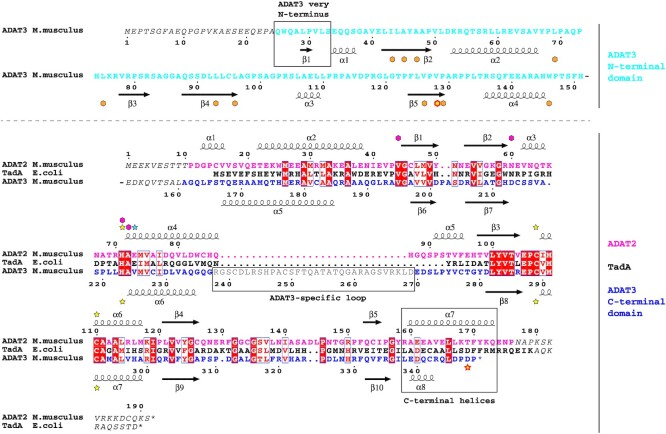
Sequence alignment of mouse ADAT2 and ADAT3 subunits and *Escherichia coli* TadA. The alignment of the sequences of mouse (M.musculus) ADAT2 (pink) and ADAT3 (N-terminal domain, cyan; C-terminal domain, blue) subunits and of *E. coli* TadA (black) shows an overall conservation of their deaminase domain (lower part of alignment) which extends to their secondary structure elements. ADAT3 has diverged from ADAT2 and TadA, including a specific N-terminal domain (cyan) and a shorter C-terminal α-helix (boxed). Residues not observed in density are italicized and the ADAT3-specific loop removed for crystallization is shown in grey and boxed. Numbering as well as secondary structure elements for mouse ADAT2 and ADAT3 are shown above and below the alignment, respectively. Yellow stars: zinc binding residues. The fourth zinc ligand in ADAT3 is represented by a red-framed yellow star. Blue star: TadA (and potentially ADAT2) glutamate involved in proton shuttling. Purple diamonds: TadA (and potentially ADAT2) residues involved in wobble adenine binding. Orange diamonds: ADAT3 residues participating in ADAT3 V128 (red circled yellow diamond) hydrophobic core. The sequence alignments were produced with Espript (48).

In eukaryotes, the enzyme responsible for wobble adenosine 34 modification into inosine is the essential ADAT (ADAT2/ADAT3; Tad2p/Tad3p in yeast) heterodimeric complex (18–22). Both ADAT2 and the C-terminal domain of ADAT3 show sequence homology to the TadA enzyme (Figure 1). Yet, whereas ADAT2 appears to have conserved TadA residues required for activity, ADAT3 has been suggested to be inactive, the essential glutamate proposed to be involved in proton shuttling during catalysis being replaced by a valine in ADAT3 (18,22). In yeast, mutation of this valine into glutamate in Tad3p could not rescue the loss of activity of a Tad2p mutant where the equivalent glutamate was changed into alanine (18). Therefore, ADAT2 and ADAT3 play different roles in the deamination reaction by the ADAT complex. Additionally, in contrast to TadA, the ADAT heterodimeric complex requires a full tRNA to perform its deamination reaction. Specifically, both the tRNA tertiary structure and the tRNA strict positioning are essential for ADAT activity (14,23,24).

Another major difference with prokaryotic TadA is the presence in ADAT3 of an additional N-terminal domain (Figure 1) whose function has so far remained elusive. In humans, mutation of valine 128 into methionine (p.Val128Met) in this N-terminal domain causes intellectual disability, microcephaly, strabismus and several other neurodevelopmental abnormalities (25–28). In cell lines derived from affected individuals, the p.Val128Met mutation (mutant hereafter termed V128M; also described as p.Val144Met in a N-terminally 16 amino acids longer human transcript) is responsible for reducing the levels of inosine at the wobble position of cognate tRNAs (29). Mutant ADAT3 alone displays an increased propensity to aggregate and to associate with protein chaperones, but its heterodimerization with ADAT2 is not perturbed, only the tRNA deamination activity of the ADAT V128M mutant complex being affected (29).

Here we have solved the crystal structure of the mouse ADAT2/ADAT3 complex. Our structure and associated enzymatic and biophysical analyses reveal that while ADAT2 active site catalyses the deamination reaction, ADAT3 active site is inactivated by the replacement by a valine of the glutamate involved in proton shuttling during catalysis but also by the capping of its zinc-binding pocket and the loss of tRNA recognition determinants. Our results show that the ADAT3 N-terminal domain adopts a fold that shares similarity with the ferredoxin-like domains (FLD) of other tRNA-modifying enzymes, yet with an additional specific structural subdomain, containing V128, which forms a loose interface between ADAT3 N- and C-terminal domains. ADAT3 N-terminal domain is essential for tRNA binding and deamination by ADAT and can rotate with respect to the catalytic domain formed by ADAT2 and the ADAT3 C-terminal domain, without affecting the structure of this latter catalytic domain. Interestingly, binding and deamination levels are varying depending on the cognate tRNA, showing that each cognate tRNA interacts differently with ADAT.

In addition, we observe that the mouse ADAT2/ADAT3-V128M mutant can still bind tRNAs as strongly as the wild-type complex but shows a reduced deamination activity. Our data show that the V128M mutation perturbs but does not prevent the folding of the ADAT3 N-terminal domain. These changes most likely affect the optimal presentation of the ASL of the tRNAs to ADAT2 and lead to reduced deamination activity. *In vivo*, inactivation of ADAT2, removal of ADAT3 N-terminal domain and the ADAT3-V128M mutant cause similar defects in neuronal migration, confirming the catalytic role of ADAT2 and the functional importance of ADAT3 N-terminal domain. Collectively, our results provide the molecular basis for mammalian ADAT complex tRNAs wobble adenine to inosine deamination and shed light on the implication of ADAT in intellectual disability, microcephaly and other neurodevelopmental disorders.

## MATERIAL AND METHODS

### Molecular cloning and mutagenesis

The genes encoding for full-length mouse ADAT2 and ADAT3 (NCBI reference sequences NM_025748.4 and NM_001100606) have been amplified by polymerase-chain reaction from E16.5 cortices and cloned into bacterial co-expression vectors (30,31) between NdeI and BamHI restriction sites. Mutants were made either by nested PCR or rolling circle and cloned in the same vectors. Specifically, the *adat2* gene was inserted in the pnCS vector that does not code for any fusion tag. The *adat3* gene was inserted in the pnEA-HT3 vector, in frame with a 5′-sequence coding for an N-terminal histidine-tag, thioredoxin and a protease 3C cleavage site.

For *in vivo* studies, wild-type and mutant mouse ADAT2 and ADAT3 were further subcloned into the pCAGGs-HA vector by restriction-ligation. pCAGGS-HA was obtained from the PCAGEN vector (addgene cat. number 11160) by restriction ligation (EcoRI and BglII enzymes) and addition of the HA sequence (5′-TACCCATACGATGTTCCAGATTACGCT-3′).

### Large-scale overproduction and purification of mADAT2/ADAT3 and *E. coli* TadA

ADAT2/ADAT3 WT and mutants were produced by co-expression in *Escherichia coli* BL21(DE3) cells in LB Broth medium. Culture induction was performed at 22°C by adding final concentration of 0.5 mM of isopropyl-1-thio-β-D-galactopyranoside (IPTG) in presence of 100 μM of Zn(SO_4_)_2_. Cells were harvested, resuspended and lysed in a buffer containing 10 mM Tris-HCl pH 8.0 and 200 mM NaCl and centrifuged at 17 500 rpm for 1 h at 4°C. The supernatant was incubated with Talon Affinity resin (Clonetech). To release the his-tagged complex from the Talon resin, the sample was treated with 3C protease overnight at 4°C. The next day, ion exchange chromatography was performed with a HiTrap Q HP column (GE Healthcare) using a gradient of NaCl from 50 mM to 1 M NaCl to remove bound nucleic acids. The sample was then further purified by size exclusion chromatography in 10 mM Tris HCl pH 8.0, 200 mM NaCl and 0.5 mM TCEP on a 16/60 Superdex 200 gel filtration column (GE Healthcare). *Escherichia coli* TadA was produced and purified using the same protocol as for the ADAT complex.

### Protein crystallization

For crystallization, WT and mutant ADAT2/ADAT3 complex at 12 mg/ml was mixed with an equal volume of reservoir reagent and crystallized using the sitting drop vapor diffusion technique at 4, 20 and 27°C. All crystals grew within one week. Crystals of ADAT2/ADAT3 that diffracted up to 3.0 Å resolution were obtained using a crystallization condition containing 0.1 M HEPES pH 7.0, 7% PEG 8000 and 8% ethylene glycol. The second crystal form of ADAT2/ADAT3 that diffracted up to 2.0 Å resolution was obtained using a crystallization solution containing 0.1 M Bis-Tris-Propane pH 7.5, 18–20% PEG 3350 and 0.2 M KSCN or NaBr.

### Data collection, structure determination, model building and refinement

For data collection, the crystals were frozen in liquid nitrogen after their short transfer into a cryo-protectant solution composed of their crystallization conditions added with either 20% glycerol or 20% PEG200. Data collection was performed under cryogenic conditions on beamline PXIII at the Swiss Light Source synchrotron (SLS, Switzerland) using a 1 Å wavelength. Data sets collected were processed with XDS (32). Structure determination was made by collecting MAD data on ADAT zinc ions on the crystal form diffracting to 3.0 Å resolution. The phases obtained were sufficient to place the human ADAT2 homodimer (PDB code: 3hd1) in the electron density and to modify it by several cycles of manual building using Coot (33) and automated refinement using Phenix (34) to model mouse ADAT2 and the mouse ADAT3 C-terminal domain. The combined MAD and model phases were then used to build the ADAT3 N-terminal domain. The complete WT model was further refined by several cycles of manual building using Coot and automated refinement using Phenix. This first final model was used to solve the structures of the WT and V128L ADAT complexes in the second space group by molecular replacement, followed by several cycles of manual building using Coot and automated refinement using Phenix. All final models were validated using tools provided in Coot and Molprobity (35). Structures have been deposited in the Protein Data Bank under PDB IDs: 7nz7, 7nz8, 7nz9.

### Mass spectrometry analyses

Prior mass spectrometry analysis, 100 ng of the nucleic acids-containing fractions were digested with 20 μl of 0.1 U/μl of RNase T1 during 4 h at 50°C or digested with 50 μl of 0.01 U/μl of RNase V1 during 3 h at 37°C. The samples were then desalted using ZipTip C18 (Millipore) by several washes with 200 mM ammonium acetate and eluted with 50% acetonitrile in milliQ water and dried under vacuum.

The pellets containing the RNase digestion products were resuspended in 3 μl of milliQ water and separated on an Acquity peptide BEH C18 column (130 Å, 1.7 μm, 75 μm x 200 mm) using a nanoAcquity system (Waters). The column was equilibrated in buffer A containing 7.5 mM TEAA (Triethylammonium acetate), 7.0 mM TEA (Triethyammonium) and 200 mM HFIP (Hexafluoroisopropanol) at a flow rate of 300 nl/min. Oligonucleotides were eluted using a gradient from 15% to 35% of buffer B (100% Methanol) for 2 min followed by elution with an increase of buffer B to 50% in 20 min. MS and MS/MS analyses were performed using a Q-Tof SYNAPT G2-S from Waters. All experiments were performed in negative mode with a capillary voltage set at 2.6 kV and a sample cone voltage set at 30 V. Source was heated to 130°C. The sample was analyzed over an *m/z* range from 500 to 1500 for the full scan, followed by fast data direct acquisition scan (Fast DDA).

tRNA were identified by specific RNase T1 digestion products (36). All RNase T1 and V1 CID (Collision Induced Decay) MS/MS spectra were deconvoluted using the MassLynx software from Waters and manually sequenced by following the y and/or c series (w ions were also useful when sequencing was difficult or in order to confirm the sequence). Experimental mass of parents and fragments were compared to the theoretical mass obtained by the Mongo Oligo Mass Calculator (https://mods.rna.albany.edu/masspec/Mongo-Oligo) (37). tRNA identification was done by comparisons with the genomic sequences obtained from GtRNAdb (http://gtrnadb.ucsc.edu/) (38). Information about nucleoside modifications were obtained from Modomics (1).

### tRNA production and CY5-labelling

All tRNA genes used were synthesized as primers (Sigma) and inserted in the different vectors used. For *in vitro* expression, the tRNA sequences were cloned into the pUC19 vector between a T7 RNA polymerase promoter and a BstNI restriction site as previously described (39). The tRNAs were synthesized from the BstNI digested DNA by *in vitro* transcription using recombinant T7 RNA polymerase (40). After transcription, samples were treated with RQ1 RNAse-Free DNAse (Promega) and RNA transcripts were phenol-extracted and precipitated. Pelleted tRNAs were dissolved in water and loaded on 7 M Urea-15% acrylamide and 1 X TBE gels. After methylene blue staining, gel slices containing tRNA transcripts were cut from the gel. The tRNAs were eluted overnight at room temperature in 0.5 M ammonium acetate, 10 mM magnesium acetate, 0.1 mM EDTA and 0.1% SDS. After phenol extraction, tRNAs were ethanol precipitated and finally recovered in water. Their concentration was determined by absorbance measurements and by gel quantification. At this step, the final concentration varied between 400 and 700 ng/μl. The tRNAs were then used for deamination enzymatic assays and CY5-labelling.

For CY5-labelling, 25% DMSO (1 μl) was first added to the purified tRNA solution (3 μl), incubated 10 s at 100°C and immediately cooled on ice. The labelling reaction was performed in the presence of 10 units of Biolabs T4 RNA ligase (M0204), 1X supplied buffer, 1 mM ATP, 12% PEG6000, 0.1 mM hexamine cobalt chloride and 10 μM of pCp-CY5 (Jena Bioscience NU-1706-CY5). A ligase mixture was added to the tRNA-DMSO mix and ligation was performed in 10 μl overnight at 16°C. After labelling, unincorporated pCp-CY5 was trapped on a G-50 column. The CY5-labelled tRNAs were fractionated on 7 M Urea-15% acrylamide gel and scanned on a GE Healthcare Ettan DIGE imager system.

### Microscale thermophoresis

A serial dilution of the mouse ADAT2/ADAT3 (WT and mutants) and TadA complexes (300 μM) was mixed with a constant concentration of CY5-labeled tRNA^Val^(AAC), tRNA^Arg^(ACG), tRNA^Ala^(AGC) or tRNA^Gly^(CCC) (5–20 nM). Samples were prepared in a buffer containing 10 mM Tris-HCl pH 8.0, 100 mM NaCl, 0.5 mM TCEP and 0.05% Tween. Samples were then filled into standard capillaries and measurements were performed using a NanoTemper Monolith NT115 instrument at 80% red LED power and 20% MST power with Laser-On time 30 s and Laser-Off time 5 s. All assays were performed in triplicate.

### Enzymatic deamination assays

Deamination assays were done in deamination buffer (10 mM Tris-HCl pH 8.0, 100 mM NaCl, 1 mM MgCl_2_, 2 mM dithiothreitol (DTT)) using 2 μM of tRNA transcript and 5.6, 0.56 or 0.056 μM of purified enzyme complex (tRNA:protein ratios of approximately 1:3, 1:0.3 and 1:0.03) in a final volume of 5 μl. The reaction was initiated when adding the purified enzyme complex to the reaction mixture and immediately incubated at 37°C during 10 min. After incubation, the reaction was immediately stopped by phenol-chloroform extraction. The supernatant was precipitated, and the pellet containing the tRNA transcript was dissolved in 20 μl of water. The cDNA was synthesized using the SuperScript™ IV Reverse Transcriptase (Invitrogen Cat.# 18090010) according to the manufacturer’s instructions with 2 μl of the tRNA transcript solution and 0.1 μM of gene-specific complementary primer. The tRNAs were then amplified using the GoTaq® G2 Flexi (Promega Cat.# M7801) according to the manufacturer’s instructions with 5 μl of cDNA, 2.5 mM of MgCl_2_ and 1 μM of gene-specific primers. As PCR fragments were too small for direct sequencing, they were ligated using pGEM®-T Easy Vector System (Promega Cat.# A1360) according to the manufacturer’s instructions. The ligated PCR products were then reamplified using the vector-specific primer and the tRNA-specific primer. The resulting PCR fragments were precipitated, redissolved and directly sequenced with the vector-specific primer. The A-to-I deamination analysis was done by measuring peak areas at the expected nucleotide position on the sequencing electropherograms. Deamination reaction was visualized by the presence of a guanosine peak at the adenosine peak position since reverse transcriptase incorporates a cytosine in the place of inosine. Due to inherent sequencing background, guanosine peaks with areas <5% of the total areas might not fully reflect a deamination activity. Therefore, a 5% threshold has been applied when displaying the results graphically. The forward primers used for PCR were as follows (5′→3′): tRNA^Val^(AAC), GTTTCCGTAGTGTAGTGGTTATC; tRNA^Ala^(AGC), GGGGAATTAGCTCAAATGGTA; tRNA^Arg^(ACG), GGGCCAGTGGCGCAATGGA; tRNA^Gly^(ACC), GCGCCGCTGGTGTAGTGG. The reverse primers used for RT-PCR were as follows: tRNA^Val^(AAC), TGGTGTTTCCGCCCGGTTTC; tRNA^Ala^(AGC), TGGAGAATGCGGGCATCGAT; tRNA^Arg^(ACG), TGGCGAGCCAGCTAGGAGT; tRNA^Gly^(ACC), TGGTGCGCCGCCCGGG. The forward primer for the pGEM®-T Easy Vector was as follows (5′→3′): GTAAAACGACGGCCAG.

### Mice, *in utero* electroporation and brain processing and analysis

All animal studies were conducted in accordance with French regulations (EU Directive 86/609 – French Act Rural Code R 214–87 to 126) and all procedures were approved by the local ethics committee and the Research Ministry (APAFIS#15691-201806271458609). Mice were bred at the IGBMC animal facility under controlled light/dark cycles, stable temperature (19°C) and humidity (50%) conditions and were provided with food and water ad libitum.

Timed-pregnant wild-type (WT) CD1 (Charles River Laboratories) mice were anesthetized with isoflurane (2 l per min of oxygen, 4% isoflurane in the induction phase and 2% isoflurane during surgery operation; Tem Sega). The uterine horns were exposed, and a lateral ventricle of each embryo was injected using pulled glass capillaries (Harvard apparatus, 1.0OD*0.58ID*100 mml) with Fast Green (1 μg/μl; Sigma) combined with different amounts of DNA constructs using a micro injector (Eppendorf Femto Jet). We injected 0.02 μg/μl of WT or mutant pCAGGS-HA-ADAT3 constructs together with 1 μg/μl of empty NeuroD-IRES-GFP vector (41) and 0.02 μg/μl of WT or E73A pCAGGS-ADAT2 constructs at E14.5. pCAGGs-HA-empty was used as a control at 0.04 μg/μl. Plasmids were further electroporated into the neuronal progenitors adjacent to the ventricle by discharging five electric 40 volts pulses for 50 at 950 ms intervals using electrodes (diameter: 3 mm; Sonidel CUY650P3) and ECM-830 BTX square wave electroporator (VWR international). After electroporation, embryos were placed back in the abdominal cavity and the abdomen was sutured using surgical needle and thread. For E18.5 analysis, pregnant mice were sacrificed by cervical dislocation four days after surgery.

E18.5 animals were sacrificed by head sectioning and brains were fixed in 4% paraformaldehyde (PFA, Electron Microscopy Sciences) diluted in phosphate-buffered saline (PBS, HyClone) overnight at 4°C. Vibratome section were prepared as follows: after fixation, brains were washed and embedded in a 4% low-melting agarose solution (Bio-Rad) and cut at a thickness of 60 μm coronally using a vibrating-blade microtome (Leica VT1000S, Leica Microsystems). Sections were kept in PBS-azide 0.05% for short-term storage or in an antifreeze solution (30% ethyleneglycol, 20% glycerol, 30% DH2O, 20% PO4 buffer) for long-term storage. For immunolabelling, vibratome sections were permeabilized and blocked with blocking solution (5% Normal Donkey Serum (NDS, Dominic Dutscher), 0.1% Triton-X-100 in PBS) for 1 h at room temperature (RT). Sections were then incubated with anti-GFP primary antibody (Abcam, ref GFP-1020) diluted in blocking solution overnight at 4°C and with A488-coupled secondary antibody (Thermofisher, ref A-11039) and DAPI (dilution 1/1000, 1 mg/ml Sigma) diluted in PBS 0,1% Triton for 1 h at RT. Slides were mounted using Aquapolymount mounting medium (Polysciences Inc).

Images were acquired using a TCS SP8 X (Leica microsystems) confocal microscope using a 20× DRY HC PL APO CS2 objective. For all experiments, a Z-stack of 1.50 μm was acquired. Image analysis was done using ImageJ software (NIH). Cell counting was performed in 4–11 different brain sections of at least four different embryos per condition. Only similarly electroporated regions were considered for further analysis. Cortical areas (upper cortical plate, lower cortical plate, intermediate zone, subventricular zone/ventricular zone) were delimited based on cell density (nuclei count with DAPI staining) using equivalent sized boxes. Number of GFP-positive cells was determined in each cortical area to establish the percentage of positive cells. Statistical analyses were performed using GraphPad Prism 6 (GraphPad) and are represented as mean ± S.E.M. The level of significance was set at *P*<0.05. Statistical tests used and n size numbers are shown in the figure legend.

### Cell culture, transfections, protein extraction and western blot

N2A mouse neuroblastoma cells were cultured in Dulbecco’s modified Eagle’s medium (DMEM, GIBCO) with 10% foetal calf serum (FCS), penicillin 100 U/ml and streptomycin 100 μg/ml in a humidified atmosphere containing 5% CO_2_ at 37°C. Cells were transfected using Lipofectamine 2000 (Invitrogen) with 1 μg of pCAGGs-HA-ADAT3 constructs together with 1 μg of pCAGGs-ADAT2 constructs. Forty-eight hours post-transfection expression, proteins were extracted as follows: cells were lysed in RIPA buffer (50 mM Tris pH 8.0, 150 mM NaCl, 5 mM EDTA pH 8.0, 1% Triton X-100, 0.5% sodium deoxycholate, 0.1% SDS) supplemented with EDTA-free protease inhibitors (cOmplete™, Roche) for 30 min, then cells debris were removed by high speed centrifugation at 4°C for 25 min. Protein concentration was measured by spectrophotometry using Bio-Rad Bradford protein assay reagent. Samples were denatured at 95°C for 10 min in Laemmli buffer (Bio-Rad) with 2% β-mercaptoethanol, then resolved by SDS–PAGE and transferred onto nitrocellulose membranes. Membranes were blocked in 5% milk in PBS buffer with 0.1% Tween (PBS-T) and incubated overnight at 4°C with the anti-HA (Merck, ref. 11867423001; 1:1000), anti-ADAT2 (Proteintech, 13621-1-AP) or α-tubulin (Sigma, T9026) primary antibody in blocking solution. Membranes were washed three times in PBS-T, incubated at room temperature for 1 h with HRP-coupled secondary antibodies (ThermoFisher Sc.) at 1:10 000 dilution in PBS-T, followed by three times PBS-T washes. Visualization was performed by quantitative chemiluminescence using SuperSignal West Pico PLUS Chemiluminescent Substrate (Sigma). All immunoblot experiments consisted of at least three independent replicates.

## RESULTS

### Crystallization and structure determination of the mouse ADAT complex

We have reconstituted mouse ADAT2/ADAT3 by producing the complex in *E. coli* using multi-expression (30,31). The complex could be readily produced and purified by combining affinity purification and size-exclusion chromatography. Crystallization attempts were successful but the crystals did not diffract at high resolution. The addition of an ion exchange chromatographic step enabled the separation of the complex from bound nucleic acids, but crystallization attempts to obtain well-diffracting crystals remained unsuccessful.

Multiple sequence alignment of ADAT2, TadA and ADAT3 revealed that ADAT3 contains a poorly conserved region which is neither present in ADAT2 nor TadA (ADAT3-specific loop; Figure 1; Supplementary Figure S1). Therefore, an ADAT3 mutant lacking this loop was created. The ADAT2/ADAT3-Δloop mutant complex could easily be produced and purified using the same experimental procedure developed for the wild-type (WT) complex, including the ion exchange chromatography step to remove the bound nucleic acids, and further crystallized.

Two crystal forms were obtained, diffracting up to 2.1 and 3.0 Å, respectively. The mouse ADAT2/ADAT3 complex structure was solved by performing MAD phasing on the zinc ions and positioning the structure of the human ADAT2 homodimeric complex (PDB code 3hd1) within the MAD density. The models for both structures were further built through several rounds of manual building and refinement. Both models show good data collection and refinement statistics (Supplementary Table S2).

### Domain organization of the mouse ADAT complex

**Figure 2.**
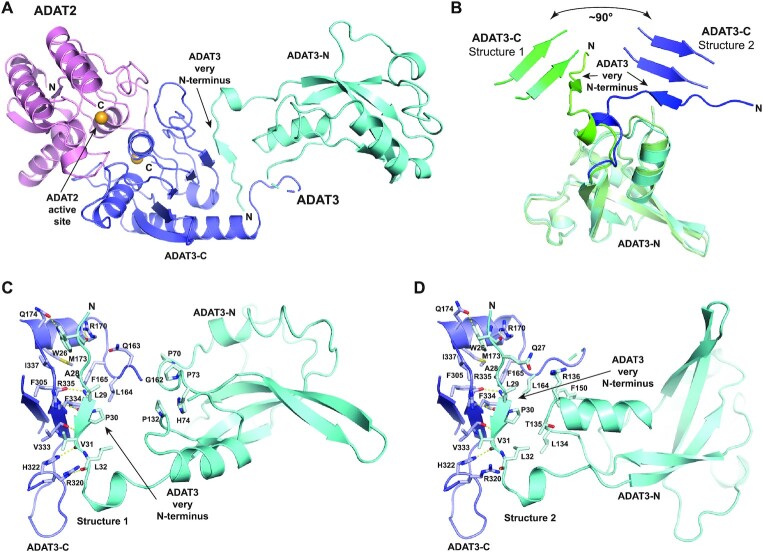
Structure of the mouse ADAT2/ADAT3 complex. (**A**) Ribbon representation of the 2.1 Å resolution structure of the mouse ADAT complex. ADAT2 is shown in pink, while ADAT3 N- and C-terminal domains are shown in cyan and blue, respectively, using the same colour code as in Figure 1. The zinc ions of both subunits are shown as orange spheres. ADAT2 active site and ADAT3 very N-terminus, which participates to the central β-sheet of ADAT3 C-terminal domain, are indicated. (**B**) Superposition of ADAT3 N-terminal domains from both structures. The different positioning of ADAT3 very N-terminus, which is linked to ADAT3 C-terminal domain, reveals that ADAT3 N- and C-terminal domains can rotate by at least 90° with respect to each other. (**C** and**D**) Close-ups of the ADAT3-N and ADAT3-C interfaces in both ADAT structures. The residues that participate in the loose interactions between the two domains are shown as sticks and labelled. Interactions between ADAT3 very N-terminus (residues 25–33) and ADAT3-C are shown, demonstrating the tight and stable interface between these two regions.

For ADAT2, only the first 10 and the last 15 residues are not seen in the electron density, while for ADAT3 the first 24 residues and residues 152–160 are not observed in the density (Figure 1). Our structures reveal that the ADAT2/ADAT3 complex harbours two semi-independent domains. The first domain (hereafter termed catalytic domain) comprises not only ADAT2 (residues 11–176) and the ADAT3 C-terminal domain (residues 161–349; hereafter termed ADAT3-C), but also ADAT3 very N-terminus (residues 25–33) that stably interacts with ADAT3-C by participating to its central β-sheet (Figures 1 and 2A).

The second ADAT domain comprises the ADAT3 N-terminal domain without the very N-terminus (residues 34–151; hereafter termed ADAT3-N) (Figure 2A). Surprisingly, the ADAT3-N domain is positioned differently in both structures with respect to the catalytic domain. Specifically, while the very N-terminus of ADAT3 interacts similarly with ADAT3-C in both structures, we observe a rotation of the ADAT3-N domains by around 90° with respect to each other (Figure 2B). This rotation is only due to the movement of residues 34–39, the rest of ADAT3-N superposing very well, and possibly of residues 152–160 that are not seen in the electron density. Thus, our structures reveal the ability of ADAT3-N to rotate with respect to the catalytic domain, even though it is doubly bound with this catalytic domain at its N- and C-termini. Yet, in both structures, ADAT3-N can still interact, albeit loosely, with the catalytic domain (Figure 2C and D).

### Molecular basis of ADAT3 catalytic inactivity

**Figure 3.**
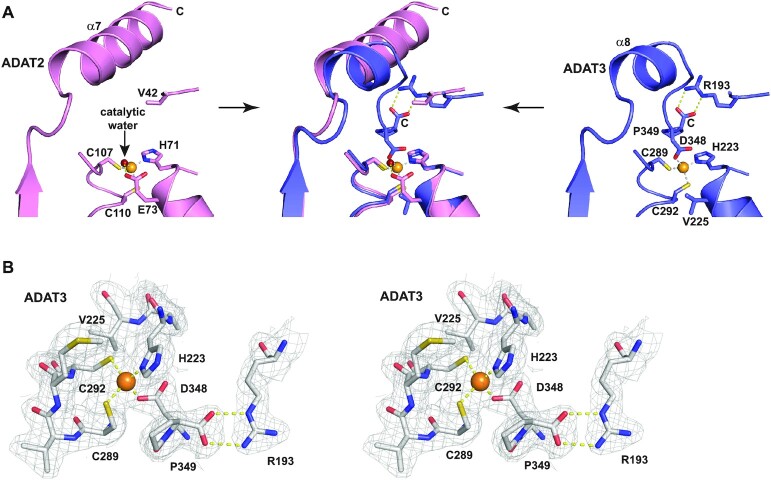
Molecular basis of ADAT3 inactivity. (**A**) Close-up view of the ADAT2 (left panel) and ADAT3 (right panel) zinc-binding pockets and their superpositions (middle panel). Zinc ions are shown as orange spheres. While ADAT2 (left panel) shows a canonical zinc coordination, including a catalytic water (red sphere), ADAT3 (right panel) has a fourth protein ligand (D348, the before last ADAT3 residue) that replaces the catalytic water. Capping of the zinc-binding pocket by the last ADAT3 residue, P349, as well as replacement of the catalytic glutamate by a valine (V225) in ADAT3 further provide the molecular basis of ADAT3 inactivation. These different interactions within the zinc-pocket of ADAT3 are allowed by the shortening of ADAT3 α8 helix compared to the longer α7 helix of ADAT2 (middle panel). (**B**) Wall-eye stereo view of ADAT3 zinc binding site. The 2Fo-Fc electron density is contoured at 1σ.

ADAT3 has been suggested to be inactivated due to the replacement by a valine of the glutamate in its zinc-binding pocket that is usually involved in proton shuttling during catalysis (18). Our structure confirms this replacement since ADAT3 V225 is positioned precisely where the equivalent ADAT2 E73 is located, V255 side chain being however turned away from ADAT3 zinc-binding pocket (Figure 3A). Yet, a zinc ion is found in ADAT3 at the same position as in ADAT2, being coordinated by a canonical triad composed of one histidine (ADAT3 H223) and two cysteines (ADAT3 C289 and C292) (Figure 3A and B).

Strikingly, however, we observe that the fourth zinc coordination is not provided by a water molecule, which in TadA, and presumably in ADAT2, participates in the deamination reaction, but by the side chain carboxylate of D348, the before last residue of ADAT3 (Figure 3A and B). Specifically, D348 carboxylate is positioned where the catalytic water is located in TadA and ADAT2. In addition, the C-terminal carboxylate of the last ADAT3 residue, P349, forms a bidentate interaction with the side chain of ADAT3 R193. This latter interaction firmly positions the proline ring as a cap over the ADAT3 zinc binding site (Figure 3A and B).

**Figure 4.**
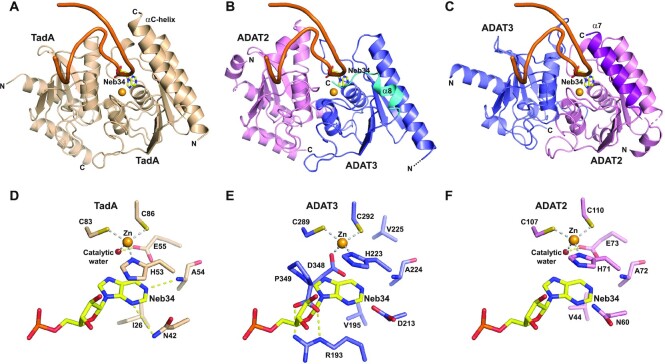
ADAT2 but not ADAT3 can accommodate an anticodon stem-loop in its active site. (**A**) Structure of an anticodon-stem-loop (ASL) bound to *S. aureus* TadA (PDB code: 2b3j) (17). The ASL is shown as an orange ribbon and the catalytic zinc as a light orange sphere. The non-hydrolysable adenine analog, nebularine, at the wobble position is shown as sticks and labeled (Neb34). The αC-helix of TadA is required for ASL binding. (**B**) Model based on our structures and the structure shown in (A) of an ASL binding to mouse ADAT in the ADAT3 zinc-binding pocket. The shorter α8 helix and the position of the ADAT3 C-terminus are incompatible with the ASL binding. (**C**) Same as in (B) but with the ASL binding in the ADAT2 active site. The ASL and the wobble adenosine could be recognized, provided minor conformational rearrangements of the ADAT complex. ADAT2 long α7 helix could participate in ASL binding. (**D**) Close-up from (A) of the interaction of wobble Nebularine within TadA. The zinc and catalytic water are shown as light orange and red spheres, respectively. (**E**) Close-up from (B) of the putative interaction of wobble Nebularine within ADAT3 zinc-binding pocket. Binding is incompatible with the position of ADAT3 C-terminus, notably aspartate 348 and C-terminal proline 349. (**F**) Close-up from (C) of the putative interaction of wobble Nebularine within ADAT2 active site. TadA and ADAT2 share the same recognition determinants of the wobble base.

These features are not observed in TadA where its long C-terminal α-helix (αC-helix), also observed in ADAT2 (α7), is not in close vicinity to the zinc ion but is essential for the binding of the ASL (Figures 1, 3A and 4A–C). In contrast, in ADAT3, the equivalent α-helix (α8) is much shorter and the last ADAT3 residues (343-349), which are not part of this helix, tightly pack in the vicinity and on top of the zinc-binding pocket (Figures 1, 3A and 4A–C).

Since TadA has been shown to accommodate five anticodon loop bases in specific pockets (17), we have looked at the possible recognition of an ASL by ADAT3 by superposing its zinc-binding site onto the TadA/ASL active site (Figure 4A and B). As expected, we observe that ADAT3 could not accommodate an ASL due to the capping of the zinc binding site by C-terminal residues that closes the pocket where the wobble adenosine should be bound. Specifically, the ADAT3 D348 side chain overlaps partially with the position of the wobble adenine, and P349 occupies the position of the sugar moiety of the wobble adenosine in the TadA/ASL complex (Figure 4D and E).

In addition, the groove and the pockets accommodating the other bases of the anticodon loop are not found in ADAT3 and the shorter α8 C-terminal helix of ADAT3 cannot interact with an incoming ASL as observed for TadA C-terminal helix (Figure 4A and B; Supplementary Figure S2). In addition, the electrostatic potential at the surface of ADAT3 above its zinc binding pocket is negative and would be repulsive for an ASL (Supplementary Figure S2). Collectively, our results provide the molecular basis for ADAT3 catalytic inactivity.

### ADAT2 active site can accommodate an ASL

In contrast, superposition of ADAT2 active site onto that of the TadA/ASL complex (17) shows that ADAT2 active site could accommodate an ASL (Figure 4A and C). In this model, the interactions with the sugar-phosphate backbone described in the TadA/ASL structure should be mostly conserved and a pocket is found in ADAT2 that can accommodate a wobble adenosine (Figure 4D and F). Importantly, ADAT2 and TadA have retained the same organization of this wobble adenosine binding pocket. Besides the conserved coordination of the zinc through ADAT2 histidine H71 (TadA H53) and cysteines C107 and C110 (TadA C83 and C86), the zinc ion is bound to a water molecule which is perfectly positioned to participate in the catalytic reaction, being also hydrogen bound to proton shuttling E73 (TadA E55). In addition, both nitrogens N1 and N3 of adenine 34 can accept hydrogen bonds from the backbone nitrogen of ADAT2 A72 (TadA A54) and the side chain of ADAT2 N60 (TadA N42). Further, ADAT2 H71 (TadA H53) and ADAT2 V44 (TadA I26) side chains can form stacking interactions with the adenine base (Figure 4D and F).

Even though pockets involved in the recognition of the other bases of the anticodon loop, as observed in the TadA/ASL complex (17), are still present in the ADAT2 active site, the recognition determinants for these other bases appear to be changed or even partially lost between TadA and ADAT2 and even in ADAT3, since this latter protein also contributes significantly to these pockets (Supplementary Figure S3). Even if the recognition of the base pair formed by nucleosides 32 and 38 could still be carried out by ADAT2 R130 (TadA K106) and ADAT3 N328 (TadA N123), recognition of the nucleosides 33, 35, 36 and 37 by ADAT2 and ADAT3 seems to have evolved, only half of the recognition determinants of TadA being conserved in ADAT2 and ADAT3 (Supplementary Figure S3). This is however in agreement with the requirement that ADAT recognizes different ASLs with different anticodon loop sequences (Supplementary Table S1) and possibly different conformations.

### ADAT3 N-terminal domain harbours a ferredoxin-like domain

**Figure 5.**
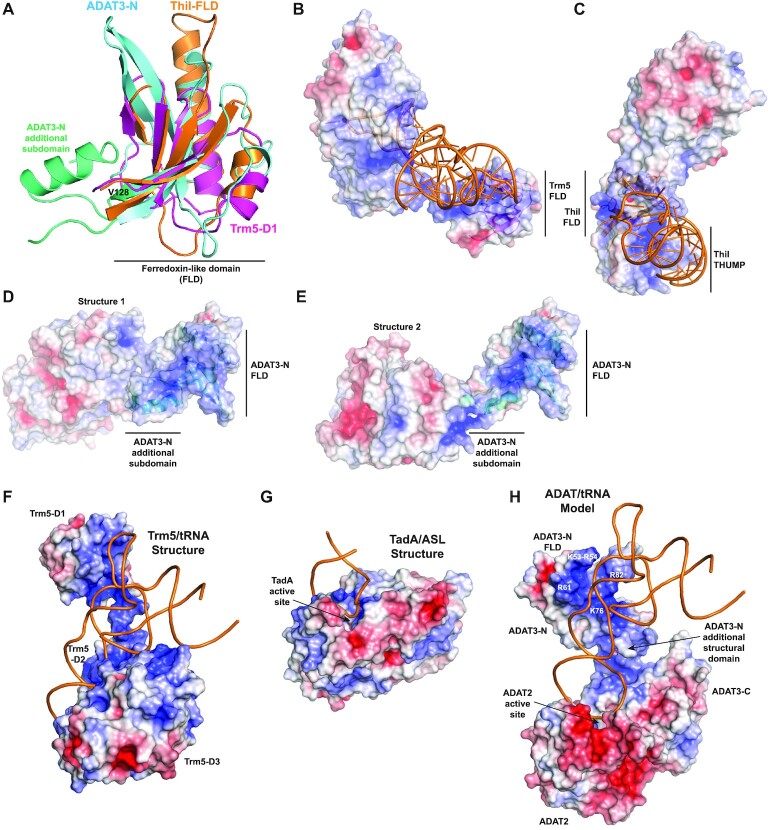
ADAT3-N harbours a ferredoxin-like domain (FLD) found in other tRNA-modifying enzymes. (**A**) Superposition of mammalian ADAT3-N, archeal Trm5-D1 (purple) and ThiI-FLD (ferredoxin-like domain; orange). All three domains adopt a FLD fold but show specific structural features. Specifically, ADAT3-N contains an additional structural subdomain (aquamarine) tightly bound to its FLD (cyan). This additional subdomain is mostly responsible for the loose interactions made between ADAT3-N and ADAT catalytic domain. (**B**) Cognate tRNA bound at the surface of Trm5. The electrostatic potential is displayed (blue, positively charged; red, negatively charged) at the surface of Trm5, showing that the tRNA interacts with the positive (blue) electrostatic patches in Trm5-FLD. (**C**) Same as in (b) for the ThiI/tRNA complex. (**D** and**E**) Electrostatic potential at the surface of the ADAT complex for both structures obtained, showing positive electrostatic patches that could interact with an incoming tRNA. For figures in (B–E), the FLDs are displayed as ribbons in the same orientation, after their superposition, showing that each FLD should interact with different surfaces with tRNAs. (**F**) Structure of archeal Trm5 bound to a cognate tRNA (PDB code: 2zzm). The electrostatic potential is represented at the surface of the Trm5 protein and the tRNA is shown as orange ribbon. The active site of Trm5 is not seen, being in the back of the enzyme. (**G**) Structure of the prokaryotic TadA/ASL complex (PDB code: 2b3j) with the same features as in (F). The orientation of the ASL is identical as in (F). The ASL makes a limited number of contacts with TadA, only nucleosides 32 to 38 interacting with the protein. (**H**) Model of an ADAT/tRNA complex based on the structures shown in (F and G), keeping the position of the ASL as in (F) and (G), and rotating slightly the position of the ADAT3-N domain from its observed position in the high resolution ADAT structure. The ASL might undergo conformational changes upon ADAT binding, as observed in the TadA/ASL complex (not included in the current model). The model shows that the ADAT3-N domain could participate to tRNA binding by interacting, notably through its FLD, with parts of the ASL stem, the D-arm and possibly, but to a lesser extent, with the variable arm of the incoming tRNAs. Basic residues changed to glutamates in ADAT3-acidic1 and ADAT3-acidic2 mutants are coloured white.

To reduce the risk of ADAT recognizing and modifying a larger set of RNA molecules in the cell due to this loss of recognition determinants, our analysis suggests that this complex has developed additional mechanisms to ensure selective modification of tRNAs. In this view, the presence in ADAT3 of a eukaryote-specific N-terminal domain is intriguing. We have used the DALI server (42) to look for structural domains potentially similar to ADAT3-N. Among the top solutions, we found the structure of the tRNA-bound archeal Trm5 (guanine-37-N1-methyltransferase) enzyme, which modifies guanosines at position 37 of tRNAs into N1-methylguanosine (43). Interestingly, we could show that the ADAT3-N domain can superpose partially with the Trm5 N-terminal D1 domain that participates in tRNA binding (Figure 5A and B). Trm5-D1 can adopt different positions with respect to the Trm5 catalytic domain, reminiscent of what is observed for ADAT3-N. This movement of Trm5-D1 is required to correctly position this domain to interact with the incoming tRNA.

Our analyses further showed that Trm5-D1 adopts a ferredoxin-like domain (FLD) fold also found in other tRNA-modifying enzymes, such as archaeal CDAT8 (cytidine deaminase acting on tRNA base C8) and prokaryotic ThiI (4-thiouridine synthetase). These latter enzymes both use a tandem FLD-THUMP domain for interacting with tRNAs (Figure 5B and C) (44,45). ADAT3-N can also partially superpose with the FLDs of these tRNA-modifying enzymes (Figure 5A). Yet, whereas Trm5, CDAT8 and ThiI FLDs form single structural domains, ADAT3-N harbours an additional structural subdomain composed of residues 37–42 and 128–151 that correspond to the N- and C-terminal extremities of ADAT3-N. Interestingly, this structural subdomain is tightly bound to ADAT3-N FLD (residues 43–127) through hydrophobic interactions and is mostly responsible for the loose interactions observed between ADAT3-N and the ADAT catalytic domain, forming a hinge between ADAT3-N FLD and ADAT catalytic domain (Figures 2C,D and 5D,E).

Analysis of the Trm5/tRNA and ThiI/tRNA structures shows that the surfaces of their FLDs which bind tRNA are different for both enzymes, the interactions being correlated with the positive electrostatic patches at the surface of their FLDs (Figure 5B and C) (43,44). This shows the functional plasticity of the FLDs in tRNA binding and suggests that ADAT3-N could also bind to tRNAs. Analysis of the electrostatic potential at the surface of ADAT3-N reveals that this domain also harbours large positive patches that could indeed be used to bind tRNAs (Figure 5D and E).

### ADAT3 N-terminal domain is required for tRNA binding by ADAT

When establishing the purification protocol for the ADAT complex, we realized that the complex co-purifies with nucleic acids that we could remove by an additional ion exchange purification step (Supplementary Figure S4a). Analysis of the nucleic acid fraction on agarose gel showed that these nucleic acids had a molecular weight around 100 bp (Supplementary Figure S4b). We sequenced by mass spectrometry the nucleic acids fraction directly obtained from the ion-exchange column. Strikingly, these were exclusively composed of tRNAs from *E. coli*, the expression host used for the production of the ADAT complex. Specifically, tRNAs with >10 different anticodons could be unambiguously assigned (Supplementary Figure S4c). The reason why ADAT selected primarily this subset of tRNAs among all *E. coli* tRNAs is unknown and could be linked to many different parameters such as growth rate, tRNA abundance, tRNA modifications but also purification conditions, among others.

These results prompted us to purify *E. coli* TadA and check whether this enzyme could also co-purify with tRNAs. Ion exchange chromatography showed indeed two peaks containing nucleic acids that were co-purifying with *E. coli* TadA. Mass spectrometry revealed that both peaks contained TadA cognate tRNA^Arg^(AGC). If the first peak was exclusively composed of this tRNA, the second peak also contained traces of degraded 16S ribosomal RNA. Strikingly, both mass spectrometry and sequencing showed that the co-purifying *E. coli* tRNA^Arg^(AGC) was in fact fully modified with inosine at the wobble position, indicating that TadA can interact stably with its product (Supplementary Figure S4d). Surprisingly, among the other modifications observed, a methylated guanosine was also found at position 18, a modification not yet reported for this *E. coli* tRNA in the MODOMICS database (1).

The results on the ADAT complex suggested that this complex can recognize and bind stably to tRNAs that do not harbour the ASL of a cognate tRNA. We, therefore, investigated the importance of the additional N-terminal domain of ADAT3 in tRNA binding. We created a mutant of ADAT3 (ADAT3-ΔN) encompassing only ADAT3-C and co-expressed it with ADAT2. A stable ADAT2/ADAT3-ΔN mutant complex was obtained that could readily be purified. However, during complex purification, almost no nucleic acids co-purified with ADAT2/ADAT3-ΔN (Supplementary Figure S4a), suggesting that ADAT3-N is important for tRNA binding by ADAT.

Since these results had been obtained with prokaryotic tRNAs, we then looked at mouse tRNA binding by mouse ADAT using microscale thermophoresis. First, cognate mouse tRNA^Val^(AAC) was produced, purified and CY5-labelled, and its binding by the ADAT complex was determined using microscale thermophoresis (MST). WT ADAT bound to tRNA^Val^(AAC) with a *K*_d_ of 3.5 ± 0.2 μM. In contrast, the ADAT2/ADAT3-ΔN mutant had a *K*_d_ of 44.5 ± 4.4 μM for the same tRNA, >10 times higher than the WT complex (Supplementary Figure S5a). Interestingly, the same difference was observed using a non-cognate tRNA^Gly^(CCC), with *K*_d_ values of 3.0 ± 0.2 μM for the WT complex and 35.8 ± 2.8 μM for the ADAT2/ADAT3-ΔN mutant (Supplementary Figure S5a). We then checked two other cognate tRNAs, tRNA^Arg^(ACG) and tRNA^Ala^(AGC). Whereas the former showed a similar *K*_d_ of 3.7 ± 0.3 μM for binding to WT ADAT, the latter had a slightly higher *K*_d_ of 8.0 ± 0.8 μM (Supplementary Figure S5b). Interestingly, TadA showed a *K*_d_ of 6.6 ± 1.1 μM for tRNA^Arg^(ACG) (Supplementary Figure S5b). Collectively, our results demonstrated the importance of ADAT3-N for tRNA binding by ADAT but showed that this domain does not discriminate between cognate and non-cognate tRNAs.

We have tentatively modelled tRNA binding by ADAT by superposing onto the TadA/ASL complex (i) our two ADAT structures and (ii) the Trm5/tRNA complex. In the former case, the catalytic dimeric domains of TadA and ADAT2/ADAT3 were used for superposition, whereas in the latter case only the ASLs of the TadA/ASL and Trm5/tRNA structures were considered. This led to two ADAT/tRNA models, one for each ADAT structure, where the ADAT3-N domain had a different position related to the tRNA molecule. Inspection of the location of the positively charged surfaces of ADAT3-N with respect to the tRNA revealed that these surfaces are completely opposite to the tRNA in the low resolution ADAT structure.

In contrast, in the case of the high resolution structure, we observed that a slight rotation of ADAT3-N could bring its positively charged surfaces in close vicinity of the tRNA backbone. In this model, both ADAT3-N and the ADAT catalytic domain are positioned relative to each other so that the two flexible ADAT3 stretches (34-39 and 152–160) could accommodate the positioning of these two domains. The final model (Figure 5F–H) shows that ADAT3-N could make extensive interactions with the upper part of the stem of the ASL and with the tRNA D arm. Interactions with other parts of the tRNA, like the variable arm, could also occur.

Specifically, our model suggests that ADAT3-N FLD could be primarily responsible for the interaction with the tRNA D arm and the upper part of the stem of the ASL, while ADAT3-N additional structural subdomain would play its role of hinge between the FLD and ADAT catalytic domain, still contributing to overall binding through its positive charge. Finally, as observed in TadA, ADAT2 active site would recognize the tRNA anticodon loop (Figure 5G and H).

### ADAT deaminates differently its cognate tRNAs and partially discriminates non-cognate tRNAs

**Figure 6.**
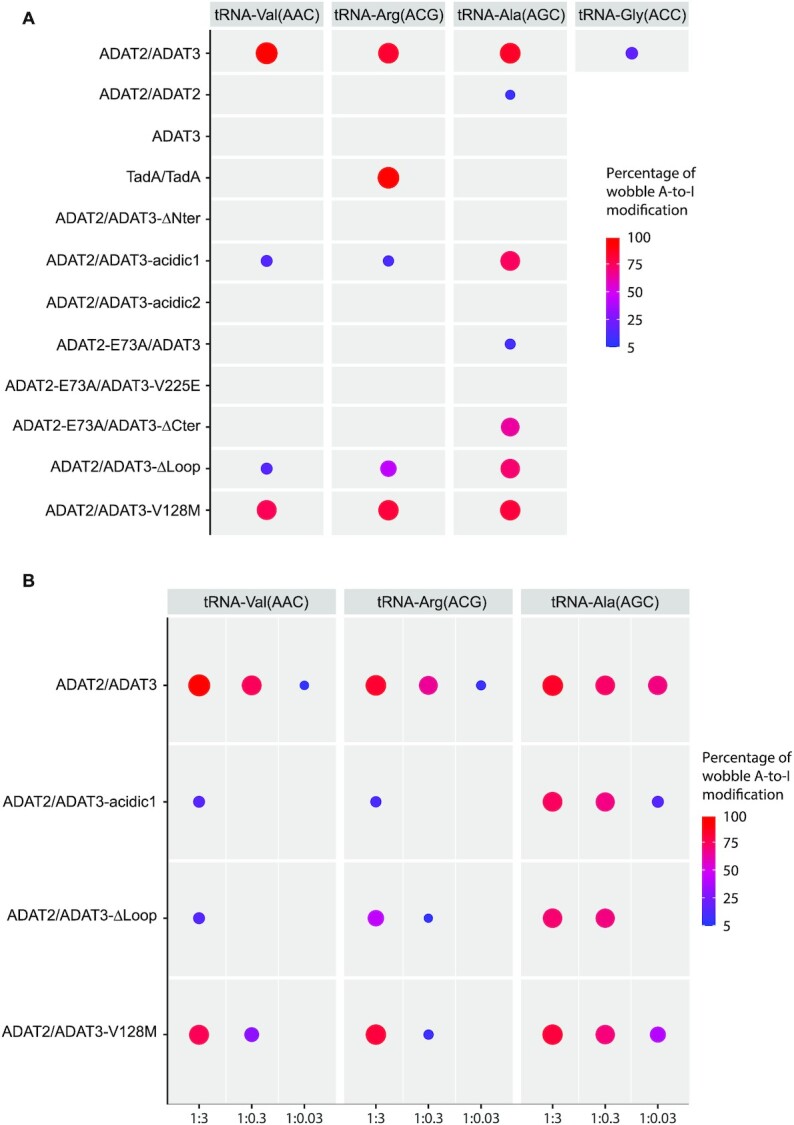
Deamination activity of ADAT, ADAT2, ADAT3 and TadA and various ADAT mutants. (**A** and**B**) The percentages of deamination activity of ADAT(ADAT2/ADAT3), ADAT2, ADAT3 and TadA and of various ADAT mutants on four different tRNAs (tRNA^Val^(AAC), tRNA^Arg^(ACG) and tRNA^Ala^(AGC), and the pseudo-cognate mutant tRNA^Gly^(ACC)) are shown as circles decreasing in size for a decreasing deamination activity. The threshold for activity has been set at 5%. The full data for all complexes is provided in Supplementary Table S3. Different tRNA:enzyme ratios (1:3, 1:0.3, 1:0.03) were used for the measurements. In (A) only the 1:3 ratio is shown for all complexes, while in (B) the three ratios are shown for a few complexes that did not appear to lose activity at high enzyme concentration but turn out to be less active when the ratio is decreasing. Only ADAT and TadA were shown to have a robust deamination activity. All enzyme mutants showed perturbation of the deamination activity, albeit to a lesser extent in the case of tRNA^Ala^(AGC) that appears to bind slightly differently to ADAT, but still requires ADAT3-N. TadA only deaminates tRNA^Arg^(ACG), and ADAT can partially deaminate a pseudo-cognate mutant tRNA^Gly^(ACC), where the wobble cytosine has been replaced by an adenosine.

In order to characterize the structure/function relationships of ADAT, we next performed deamination assays. Using initially the WT complex and different ADAT:tRNA^Val^(AAC) ratios, we observed decreasing amounts of inosine-modified tRNA for decreasing amount of ADAT complex for a fixed reaction time. Surprisingly, time-course experiments did not yield much higher tRNA modification for longer incubation times at a fixed ADAT concentration, although the enzyme appeared highly processive since most of the tRNA was modified within seconds in excess of ADAT. Since *E. coli* TadA can co-purify with its product, we reasoned that the ADAT product could also remain bound to this enzyme, at least to the ADAT3 N-terminal domain, perturbing time course assays by competing with unmodified tRNAs. We, therefore, proceeded using different ADAT:tRNA ratios and a fixed reaction time (Figure 6; Supplementary Table S3).

The three tRNA^Val^(AAC), tRNA^Arg^(ACG)and tRNA^Ala^(AGC) were used for these assays. We not only first measured the deamination activity of the WT ADAT2/ADAT3 complex, but also tested the activity of, as assessed by size exclusion chromatography, the homodimeric ADAT2/ADAT2 complex, the monomeric ADAT3 protein and the homodimeric *E. coli* TadA. WT ADAT showed a robust deamination activity, while ADAT2/ADAT2 and ADAT3 were inactive (Figure 6A). TadA homodimer was active only in presence of tRNA^Arg^(ACG), as previously shown (Figure 6A) (13). Decreasing ADAT:tRNA ratios revealed however that ADAT could more readily deaminate tRNA^Ala^(AGC) than the two other cognate tRNAs used, possibly reflecting a higher turnover rate for tRNA^Ala^(AGC) (Figure 6B; Supplementary Table S3).

We next analysed the importance of the ADAT3 N-terminal domain on activity. The ADAT2/ADAT3-ΔN mutant completely abolished deamination for the three tRNAs (Figure 6A). Since our ADAT/tRNA model suggested that incoming tRNAs could bind to positively charged patches on ADAT3-N FLD, we identified two sets of basic exposed residues that could participate in tRNA binding: K53-R54-R61 and K76-R82 (Figure 5H). We constructed two mutants where these residues were mutated into glutamates, one mutant modifying only the first set of residues (ADAT3-acidic1), the second mutant modifying both sets of residues (ADAT3-acidic2). Both complexes were unable to co-purify with *E. coli* tRNAs. Interestingly, while the ADAT-acidic1 mutant showed some residual deamination activity, notably for tRNA^Ala^(AGC), the ADAT-acidic2 mutant completely lost its deamination activity, whatever the tRNA (Figure 6A and B; Supplementary Table S3). These results therefore reinforced our modelling studies and binding hypothesis.

We next investigated the two zinc binding sites of ADAT2 and ADAT3. An ADAT2-E73A/ADAT3 mutant, where the proton shuttling residue E73 was mutated into alanine, hardly retained any activity (Figure 6A). We then tested whether ADAT3 zinc binding pocket mutants could rescue the inactivity of the ADAT2-E73A mutant. Three ADAT3 mutants were constructed: ADAT3-V225E, ADAT3-ΔC, where the capping of the ADAT3 zinc binding site was prevented by removing the last five residues (345-349) of ADAT3, and ADAT3-V225E-ΔC. Only the first two mutants turned out to be soluble upon co-expression with ADAT2. Intriguingly, while the ADAT2-E73A/ADAT3-V225E mutant was completely inactive, the ADAT2-E73A/ADAT3-ΔC mutant was inactive for tRNA^Val^(AAC) and tRNA^Arg^(ACG) but showed significant deamination activity for tRNA^Ala^(AGC), albeit only in presence of high amounts of ADAT (Figure 6A; Supplementary Table S3).

We also investigated the role of the ADAT3-specific loop that we removed for crystallization (Figure 1). Interestingly, the ADAT2/ADAT3-Δloop mutant showed a significant reduction of deamination activity, notably for tRNA^Val^(AAC) and tRNA^Arg^(ACG) (Figure 6A and B). We therefore tested the binding of this mutant to the three tRNAs by microscale thermophoresis. *K*_d_ values for the three complexes were very similar: 6.7 ± 0.7 μM (Val), 6.5 ± 0.2 μM (Arg) and 6.7 ± 0.3 μM (Ala). These values were slightly higher for tRNA^Val^(AAC) and tRNA^Arg^(ACG) but slightly lower for tRNA^Ala^(AGC) when compared to the WT complex (Supplementary Figure S5c). These results showed that the ADAT3-specific loop, which is located in the ADAT3 C-terminal domain and is close to the ADAT2 active site, can influence positively or negatively tRNA binding into ADAT2 active site.

Finally, we have asked whether ADAT could deaminate a non-cognate tRNA having an adenosine at the wobble position. Since we previously showed that tRNA^Gly^(CCC) was able to bind to ADAT, we created a mutant of this tRNA by replacing its wobble cytosine with an adenosine (tRNA^Gly^(ACC)). The anticodon loop sequence of this mutant tRNA perfectly fitted the diversity of base recognition required by ADAT2 to recognize all ADAT cognate tRNAs (Supplementary Table S1). Interestingly, WT ADAT was able to modify the wobble adenosine into inosine of the pseudo-cognate tRNA^Gly^(ACC), albeit much poorly as for a cognate tRNA (Figure 6A). This demonstrated that ADAT has still retained specificity determinants for its cognate tRNAs beside the recognition of the wobble adenine.

### The V128M mutation enlarges ADAT3-N without precluding tRNA binding but affects ADAT deamination activity

**Figure 7.**
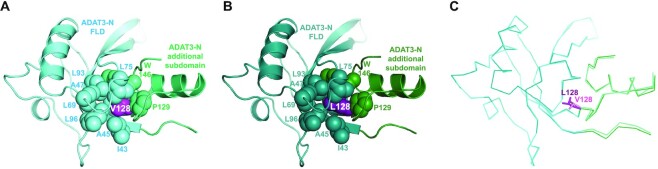
Position of V128 within ADAT3-N and effect of its mutation in leucine (V128L). (**A**) Ribbon structure of ADAT3-N with the residues forming its central V128 (purple) hydrophobic core shown as spheres. The residues are coloured according to the subdomain they belong to: FLD (cyan) and additional structural subdomain (aquamarine). (**B**) Same as (A) for the V128L mutant. The same colour code is used with slightly darker colours. (**C**) Superposition of the ADAT3-N WT and V128L domains shown as Cα ribbons. Slight changes are observed in the main chain position (movements of 0.3–0.9 Å) of residues from the V128 hydrophobic core and of neighbouring residues upon the V128L mutation. These movements propagate notably within the ADAT3-N specific structural subdomain but much less in the FLD. A V128M mutation is expected to exacerbate these changes.

Mutation of valine 128 into methionine (p.Val128Met) in human ADAT3-N has been shown to cause intellectual disability, microcephaly and other neurodevelopmental disorders (25–28). We observe that the equivalent valine 128 in mouse ADAT3 is part of a large hydrophobic core comprising I43, A45, A47, L69, L75, L93, L96, V126, P129 and W146 (Figures 1 and 7A). This hydrophobic core is located in the centre of ADAT3-N and is responsible for the tight interaction between the ADAT3-N FLD and the additional structural subdomain of ADAT3-N. This suggested that the V128M mutation would affect the overall folding of ADAT3-N. To test this hypothesis, we generated constructs bearing the V128M mutation but also the V128L or V128I mutations, those latter two substitutions having potentially a milder effect on ADAT3-N folding.

All three ADAT3 mutants could be co-expressed with ADAT2 and their complex purified to homogeneity following the same experimental protocol as for the WT enzyme. All mutant complexes co-purified with nucleic acids (Supplementary Figure S4a,b). We showed by mass spectrometry that these nucleic acids were also composed of diverse *E. coli* tRNAs, as for WT ADAT (Supplementary Figure S4c). Interestingly, all three mutants could be crystallized in similar conditions as for the WT complex, albeit with some decrease in crystallization propensity for the V128M mutant complex. The tendency of the crystals to aggregate, as already observed with the WT complex, was also observed with the mutants and hindered our structural analyses. Whereas the crystals with the V128I and V128M mutants, which showed stronger aggregation, did not diffract sufficiently, we were able to collect a full data set at 2.0 Å resolution for the V128L mutant. The structure of the ADAT-V128L complex was refined with good data collection and refinement statistics (Supplementary Table S2).

The same crystallographic space group and unit cell as well as the similar resolution obtained for both WT and V128L ADAT complexes allowed the precise analysis of the structural changes occurring upon the V128L mutation. Superposition of the complexes showed that both ADAT3-N and the ADAT catalytic domain are mostly unaffected by the mutation. In details, however, we observed that the main chain of residues involved in the V128 central hydrophobic core and of their neighbours is moving away from the core by about 0.3–0.9 Å (Figure 7A–C). We found that these movements are mainly propagated within the ADAT3-N specific structural subdomain but much less in the FLD, which displays a more stable fold due to its tighter packing (Figure 7C).

A bulkier methionine in place of the leucine at position 128 will have a more substantial effect on the ADAT3-N structure and we expect the movements observed in the case of the V128L mutation to be larger in the V128M mutant. Yet, since the ADAT-V128M complex can crystallize in conditions where the ADAT3-N domain participates in crystal packing, it appears unlikely that the movements induced by the V128M mutation would cause the complete unfolding of the ADAT3-N domain. We have assessed the enzymatic activity of the ADAT-V128M mutant and showed that it retains a significant activity. However, this occurs notably when the enzyme is in excess compared to the tRNA. In addition, this activity is always lower than that of the WT enzyme at the same concentration (Figure 6A,B; Supplementary Table S3). Of note, we did not observe that this mutant modified any other adenosines in these tRNAs, showing that, at least for the tRNAs considered, the V128M mutation does not cause a loss of specificity for the targeted adenosine.

Since the V128 mutants co-purified with *E. coli* tRNAs, we also looked by microscale thermophoresis at tRNA binding by the mutants to the cognate tRNA^Val^(AAC). Our measurements gave *K*_d_ values of 3.3 ± 0.2, 4.2 ± 0.4 and 2.5 ± 0.1 μM for the V128I, V128L and V128M mutants, respectively (Supplementary Figure S5d). These quantitative results show minimal differences for tRNA binding compared to the WT, in agreement with the qualitative observations made with the *E. coli* tRNAs during complex purification. As such, loss of tRNA binding by the V128M cannot be considered as a major reason for the reported decrease of tRNA deamination observed *in vitro* (Figure 6A and B) and in patient cells (29), implying that this mutation perturbs other mechanisms.

### Mutant ADAT complexes show impaired neuronal migration *in vivo*

**Figure 8.**
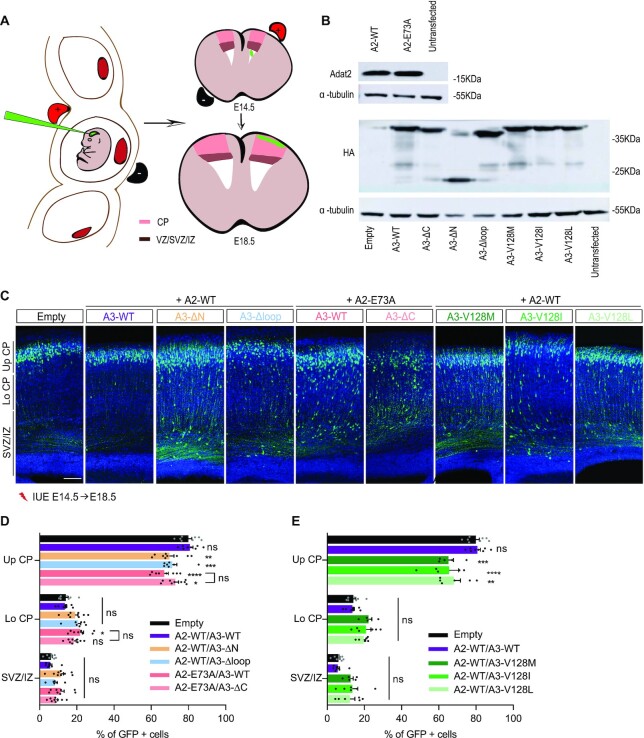
*In vivo* effects of mutant ADAT complexes. (**A**) Scheme representing the *in utero* electroporation procedure used to follow the migration of GFP+ neurons expressing WT and mutant ADAT2 and/or ADAT3. (**B**) Western blot of extract from N2A cells transfected with an empty vector or the indicated ADAT2 (upper panel) or HA-tagged ADAT3 (lower panel) constructs showing similar expression of WT and catalytically inactive ADAT2 (E73A) proteins and of WT, mutant and truncated ADAT3 proteins. α-Tubulin was used as a loading control. (**C**) Coronal sections of E18.5 cortices, 4 days after IUE with NeuroD-IRES-GFP and the indicated ADAT2 and ADAT3 constructs, showing impaired distribution of GFP-positive electroporated cells (green) in all conditions tested. Nuclei are stained with DAPI (blue); scale bar: 100 μm. (**D** and**E**) Histograms (means ± S.E.M) showing the distribution of GFP-positive neurons in different regions (Up CP, Upper cortical plate; Lo CP, Lower cortical plate; IZ, intermediate zone; SVZ, subventricular zone) for all conditions as indicated. Significance was calculated by two-way ANOVA (Bonferroni’s multiple comparisons test). Number of embryos analysed: empty vector and A2-WT/A3-WT, *n* = 8; A2-WT/A3-V128L and A2-WT/A3-V128I, *n* = 6; A2-WT/A3-V128M, *n* = 5; A2-WT/A3-Δloop, A2-E73A/A3-WT and A2-E73A/A3-ΔC, *n* = 8; A2-WT/A3-ΔN, *n* = 9; ns, non-significant; *P < 0.05; **P < 0.01; ***P < 0.001; ****P < 0.0001. A2 and A3 stand for ADAT2 and ADAT3, respectively.

To evaluate the functional impact of the ADAT3-N domain *in vivo* and considering the implication of the p.Val128Met variant in neurodevelopmental disorders, we assessed the consequences of overexpressing mouse ADAT3 mutants together with ADAT2 *in vivo* in the mouse developing cortex using *in utero* electroporation (IUE) (Figure 8A). Since ADAT plays a crucial role in the regulation of migration of the projection neurons (Del-Pozo-Rodríguez, in preparation), we investigated the effects of the mutated constructs on neuronal positioning. In addition, since other mutants used in our study showed significant effects on tRNA binding and deamination, we extended our *in vivo* investigations to the most significant ones. We therefore considered seven mutants: ADAT2-E73A/ADAT3, ADAT2-E73A/ADAT3-ΔC, ADAT2/ADAT3-Δloop, ADAT2/ADAT3-ΔN, ADAT2/ADAT3-V128I, ADAT2/ADAT3-V128L and ADAT2/ADAT3-V128M.

We first verified by transfection experiments in a N2A neuroblastoma cell line that WT ADAT2 and ADAT3 as well as their mutants were similarly expressed (Figure 8B). We then expressed mouse WT or E73A ADAT2 and ADAT3 mutants using IUE of pCAGGS-ADAT2 and HA-tagged pCAGGS-ADAT3 constructs together with a NeuroD-IRES-GFP reporter plasmid, allowing the expression of GFP specifically in post mitotic neurons in WT mouse cortices at E14.5 (Figure 8A).

We then evaluated the effects of the mutants 4 days after IUE, when most of the GFP+ postmitotic neurons expressing full-length WT ADAT are reaching the cortical plate as in the control (Figure 8C). Interestingly, all neurons expressing the different variants accumulated in the intermediate zone, with a decrease of 15.7%, 9.3%, 10.8%, 12.7%, 18%, 14.8% and 18.4% of the cells reaching the upper cortical plate in the ADAT2-E73A/ADAT3, ADAT2-E73A/ADAT3-ΔC, ADAT2/ADAT3-Δloop, ADAT2/ADAT3-ΔN, ADAT2/ADAT3-V128I, ADAT2/ADAT3-V128L and ADAT2/ADAT3-V128M conditions, respectively (Figure 8D and E). These results demonstrated that the disease- and structure-based variants impede to similar extents the radial migration of projection neurons.

Interestingly, we observe no rescue of the catalytic ADAT2 inactivity phenotype by the ADAT2-E73A/ADAT3-ΔC mutant, reflecting the very partial rescue of the enzymatic activity of this mutant *in vitro*. In addition, these results further confirm the functional importance of the ADAT3-specific loop, whose deletion was required for the structural studies, as well as that of ADAT3 N-terminal domain. Thus, collectively, our *in vivo* results corroborate our *in vitro* analyses.

## DISCUSSION

Our analysis of the mammalian ADAT complex reveals specific features that distinguish this complex from the prokaryotic TadA homodimeric complex. While ADAT2 has retained structural determinants for the recognition of cognate tRNAs anticodon loops and the deamination activity, ADAT3 has become inactive through amino acid changes, the capping of its zinc binding pocket by ADAT3 C-terminus, and the disappearance of binding pockets for the anticodon loop nucleosides. *In vitro*, removal of ADAT3 zinc binding site capping in the ADAT3-ΔC mutant is not sufficient to confer a full deamination activity to ADAT3 and, *in vivo*, the ADAT3-ΔC mutant fails to rescue the phenotype caused by ADAT2 inactivation. In addition, the homodimeric ADAT2/ADAT2 complex and ADAT3 alone do not show any robust deamination activity. Although this could be due to an inadequate buffer, our structural analysis shows that both ADAT2 and ADAT3 residues are participating to the formation of ADAT active site. Our results therefore reinforce the generally admitted idea that ADAT2/ADAT3 is the functional biological unit for mammalian tRNA wobble adenosine-to-inosine deamination and show that ADAT2 is the catalytic subunit of this complex but requires several ADAT3 residues to be active.

The requirement for ADAT to modify different tRNAs imposes that ADAT2, in contrast to TadA, has evolved a specific anticodon loop recognition strategy to adapt to the various tRNAs with different anticodon loop sequences and possibly with different ASL conformations. This is in agreement with the apparent loss of some recognition determinants observed in the ADAT2 anticodon loop bases recognition pockets. In turn, this has constrained the ADAT complex to change to recognize specifically tRNA macromolecules, in agreement with the fact that the full tRNA tertiary structure, and not only the ASL, is required for ADAT activity (14,23,24).

Our results demonstrate that the eukaryote-specific ADAT3 N-terminal domain is essential for the strong, albeit non-selective interaction between ADAT and tRNAs. An important result of our structural studies is the discovery of the inherent mobility of the ADAT3-N domain with respect to the catalytic domain. This positional mobility of ADAT3-N appears linked to the fact that the ADAT complex binds and modifies differently various tRNAs having variable compositions and tertiary structures. Thus, the capacity of movement of ADAT3-N should be essential to facilitate the binding of the tRNAs and the strict positioning of their anticodon loop into the ADAT2 active site. Thus, the differences that we and others (24) observe in the recognition and the processing of cognate tRNAs by ADAT could be explained by different binding of those tRNAs to the ADAT active site but also onto ADAT3-N.

The apparent non-selective nature of ADAT3-N raises the question of the specific recognition of cognate tRNAs by ADAT. As for TadA, which only recognizes the anticodon loop of its cognate tRNA, notably its wobble adenosine, the specific recognition of the wobble adenosine by ADAT2 could suffice, this subunit scanning the tRNAs bound to ADAT3-N for a wobble adenosine. In agreement, our deamination assays with a pseudo-cognate tRNA^Gly^(ACC) mutant revealed that ADAT can deaminate *in vitro* a non-cognate tRNA which has an artificial adenosine at its wobble position, rendering its anticodon loop perfectly compatible with the recognition diversity required for deamination of all ADAT cognate tRNAs (Supplementary Table S1). However, the fact that the deamination level of this tRNA remains poor indicates that ADAT has also retained specific cognate tRNA recognition determinants but that these are possibly not only situated in ADAT2 anticodon loop binding pockets.

Whether these additional determinants are located at the level of ADAT3 N-terminal domain, in the ADAT3-specific loop, which we have shown to impact tRNA binding and ADAT enzymatic activity, or in other regions of ADAT2 and/or ADAT3, remains to be determined. We cannot exclude that all these domains contribute to various extents to specificity. Interestingly, our deamination experiments show that ADAT does not process equally its various cognate tRNAs. Notably, tRNA^Ala^(AGC) appears less sensitive to ADAT perturbations, especially those affecting mildly ADAT3 N-terminal domain, and differences in the binding of tRNA^Arg^(ACG) and tRNA^Ala^(AGC) by ADAT have already been reported (24). In line with this, tRNA^Ala^(AGC) interaction with ADAT shows a slightly higher *K*_d_ around 8 μM. This could suggest that ADAT interaction with tRNA^Ala^(AGC) is different than with tRNA^Val^(AAC) and tRNA^Arg^(ACG). However, removal of ADAT3 N-terminal domain or mutating several basic residues in ADAT3-N FLD basic patches completely prevents deamination of tRNA^Ala^(AGC), demonstrating that even tRNA^Ala^(AGC) requires ADAT3-N for binding in regions far away from ADAT2 active site.

The V128M mutation in ADAT3-N is known to cause intellectual disability, microcephaly and other neurodevelopmental disorders in patients due to a decreased wobble A-to-I modification by the ADAT complex (25–29). It has been suggested that this decrease could be due to the instability of mutant ADAT3 and its interaction with chaperones (29). The central position of V128 in the hydrophobic core organizing ADAT3-N could indeed affect ADAT3-N folding *in vivo*, potentially leading to ADAT3 interaction with chaperones. Yet, the differences observed in A-to-I modification of various tRNAs by mutant ADAT observed *in cellulo* (29) imply that the V128M mutation also plays a role in a correctly folded ADAT V128M mutant complex. Our observations that ADAT3-V128M associates with ADAT2 and that the resulting ADAT mutant complex can bind as strongly as WT to tRNAs and retains some deamination activity demonstrate that this mutant still possesses the propensity to fold correctly and to be partially active. This raises the question of how the V128M mutation could affect the deamination reaction of ADAT.

Our data suggest that the structural enlargement of ADAT3-N by the V128M mutation could perturb (i) the positioning of the tRNAs onto the ADAT3-N domain, (ii) the rotation of this domain with respect to the catalytic domain and/or (iii) the stable interface between these latter two domains during catalysis. The results presented here favour the hypothesis that the V128M mutation does not significantly perturb tRNA binding by ADAT3-N FLD but rather affects ADAT3-N additional structural subdomain and its role of hinge between ADAT3-N and the ADAT catalytic domain. This would perturb the optimal anticodon loop presentation to ADAT2 and lead to a decreased A-to-I modification. Yet, considering that we observe that the V128M mutant effect on deamination is different depending on the cognate tRNA, it is most likely that the mode of binding of the tRNA onto ADAT3-N will also influence the catalytic reaction.

**Figure 9.**
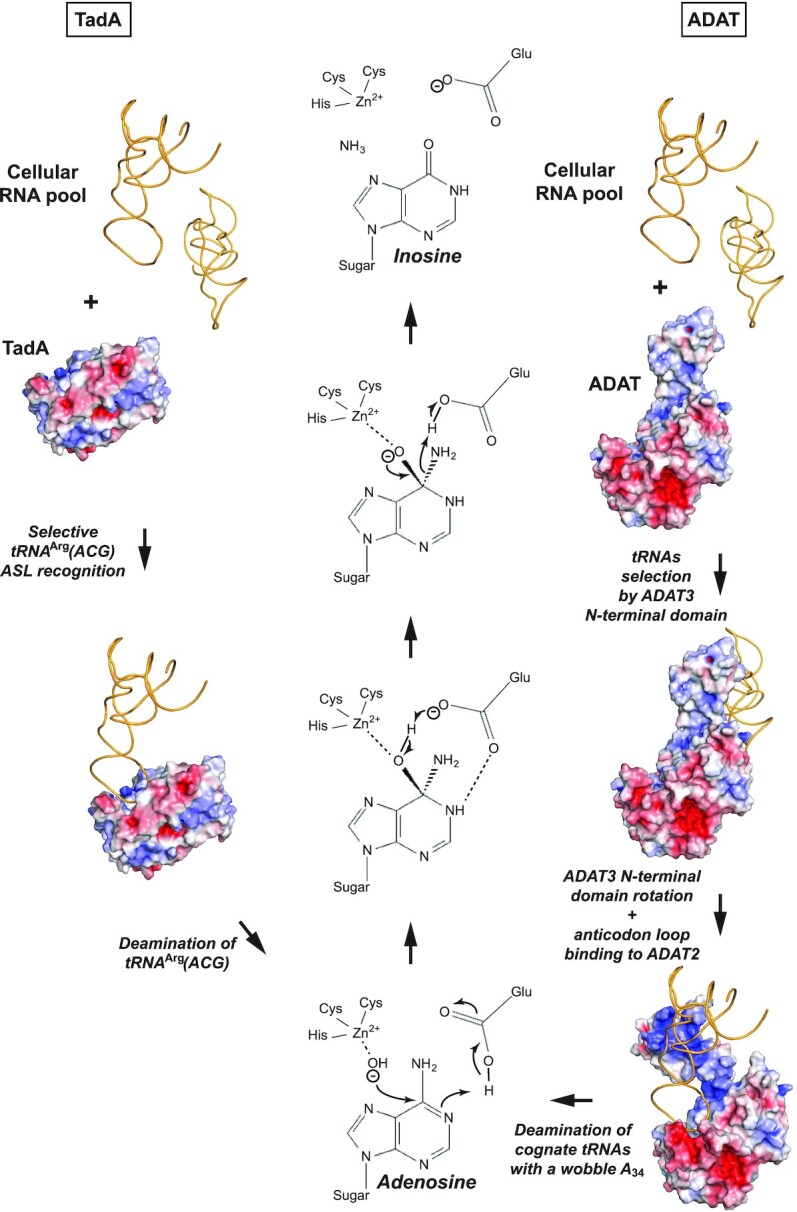
Proposed TadA and ADAT cognate tRNAs deamination mechanisms. The modes of tRNA binding and recognition for prokaryotic TadA (left) and ADAT (right) enzymes are indicated. While TadA can specifically and directly bind and recognize within its active site its cognate tRNA^Arg^(ACG) from the pool of cellular RNAs using the specific anticodon-stem-loop conformation and the anticodon loop base composition of tRNA^Arg^(ACG), ADAT appears to have evolved a two-step mechanism. ADAT first binds to tRNAs irrespective of their anticodon composition through its N-terminal domain, most likely upon recognizing the tRNAs specific three-dimensional structure. Upon rotation of ADAT3 N-terminal domain, the anticodon of the tRNAs is presented to the ADAT2 active site that recognizes its cognate tRNAs in a process requiring residues from both ADAT2 and ADAT3. Both TadA and ADAT2 deaminate the cognate tRNAs wobble adenosine into inosine using the same conserved mechanism (centre), requiring a catalytic water bound to their zinc ion as well as a proton shuttling glutamate residue.

Collectively, our results shed light on the modification of tRNAs by the ADAT complex and its similarities and difference with TadA, suggesting a two-step mechanism for the eukaryotic complex (Figure 9). First, the tRNA, especially its tertiary structure, is recognized by the ADAT3 N-terminal domain, notably its FLD. This domain then rotates to maximize the interactions between the tRNA and the full ADAT complex, and presents the ASL correctly to ADAT2 that then modifies the wobble adenosine into inosine. This mechanism is perturbed by the V128M mutation, causing a decrease in A-to-I modification, leading to neurodevelopmental disorders (Figure 9).

While this manuscript was in revision, the yeast ADAT complex structure was published (46). Conclusions of the study on the yeast complex are similar to ours, notably on the role of ADAT3/Tad3p N-terminal domain for tRNA recognition and presentation to ADAT2/Tad2p active site, as well as the inactivation of ADAT3/Tad3p by capping of its zinc binding site. Both studies, however, show significant structural differences between the yeast and mouse ADAT complexes that could relate to function (Supplementary Figure S6). Specifically, the yeast Tad2p C-terminus is much longer than that of mammalian ADAT2 and wraps around its core domain, and the ADAT3/Tad3p specific loops appear to adopt different conformations. In addition, although both ADAT3/Tad3p N-terminal domains show a similar fold, they also show significant structural differences that could impact tRNA binding, and the V128 structural environment is different in both mouse ADAT3-N and yeast Tad3p-N (Supplementary Figure S6). Surprisingly, although both mouse ADAT3 and yeast Tad3p C-termini provide the fourth zinc-ligand to this subunit, the composition and conformation of these two C-termini are quite different in both ADAT3/Tad3p subunits in contrast to the rest of their structures. Finally, the V128M equivalent mutation in yeast Tad3p causes ∼90% loss of deamination activity in presence of tRNA^Ala^. Our study shows that in similar experimental conditions (tRNA:enzyme ratio of 1:0.03), the effect of the ADAT3-V128M mutation can be more drastic, with a complete loss of activity, although we demonstrate that this effect is, in fact, varying depending on the tRNA bound. This latter aspect was not investigated in the yeast study that made use of a single tRNA species, leaving therefore open several questions on the similarities and dissimilarities of the yeast Tad2p/Tad3p mechanism compared to the vertebrate ADAT2/ADAT3 mechanism.

Thus, our study provides the molecular basis for further investigating the involvement of ADAT in diseases (25–29,47). However, the mechanisms affected by the loss of tRNA wobble A-to-I modification in disease remain to be characterized. Notably, whether faulty tRNA deamination impacts tRNAs stability, abundance, maturation and aminoacylation remains to be investigated. In addition, efficiency and/or accuracy of protein synthesis also remains to be determined. Notably, further study of actively-translated transcripts and translation rate by ribosome profiling would shed light on translational effects at the ribosome. Such studies would benefit from an analysis in the most relevant conditions, i.e. in diseased brains using knock-in or knock-out ADAT3 mouse models, to determine the consequences of ADAT variants on translation.

## DATA AVAILABILITY

The three crystallographic structures described in the manuscript have been deposited in the Protein Data Bank under the PDB IDs 7nz7, 7nz8, 7nz9.

## Supplementary Material

gkab436_Supplemental_File

## Notes

Present address: Martin Marek, Loschmidt Laboratories, Department of Experimental Biology and RECETOX, Faculty of Science, Masaryk University, Kamenice 5/A13, 625 00 Brno, Czech Republic.
