# Supplementary material for: The structure of the mouse ADAT2/ADAT3 complex reveals the molecular basis for mammalian tRNA wobble adenosine-to-inosine deamination
Source: Nucleic Acids Res. 2021 May 31;49(11):6529–48. doi: 10.1093/nar/gkab436 (PMC8216470; doi:10.1093/nar/gkab436)
Supplement: gkab436_Supplemental_File [file gkab436_supplemental_file.pdf]

The structure of the mouse ADAT2/ADAT3 complex reveals the molecular basis for mammalian tRNA wobble adenosine-to-inosine deamination

Elizabeth Ramos-Morales, Efil Bayam, Jordi Del-Pozo-Rodríguez, Thalia Salinas-Giegé, Martin Marek, Peggy Tilly, Philippe Wolff, Edouard Troesch, Eric Ennifar, Laurence Drouard, Juliette D. Godin, Christophe Romier

Supplementary information

(a) ADAT2

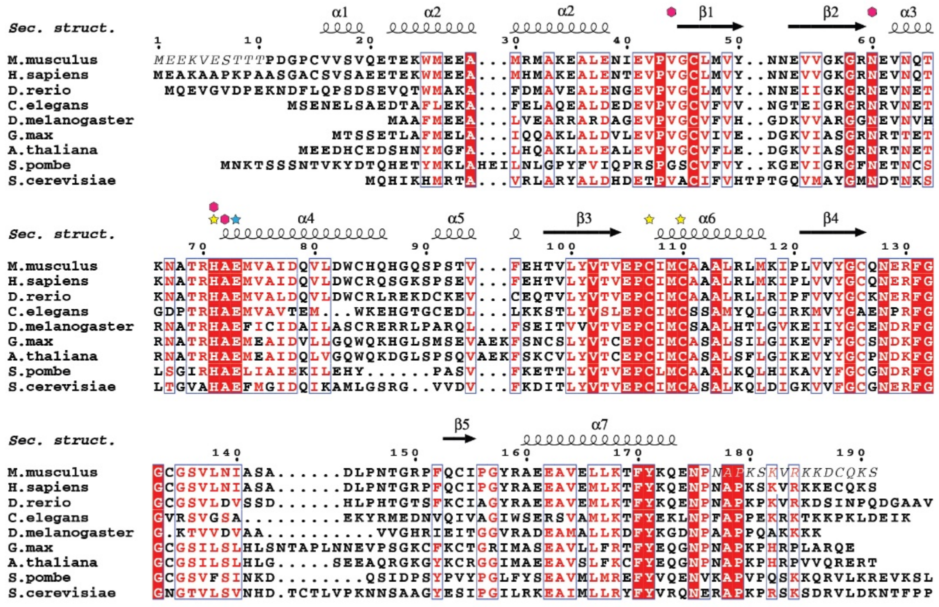

(b) ADAT3

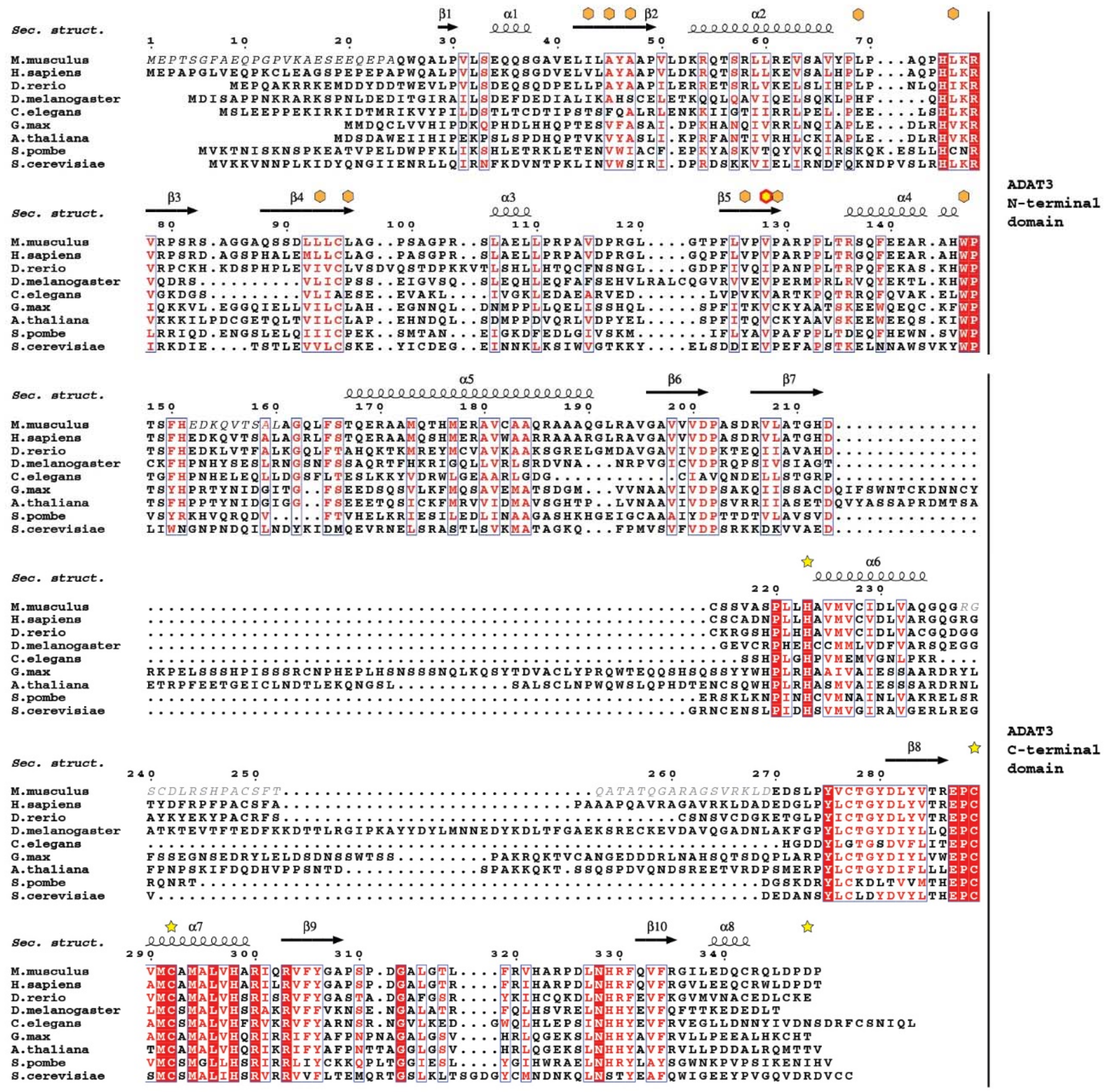

Supplementary Figure 1. Multiple sequence alignment of ADAT2 and ADAT3

(a,b) Multiple sequence alignment of (a) ADAT2 and (b) ADAT3 subunits from reference organisms. For clarity, in (a) the ADAT2 non-conserved N-terminal part of *S. pombe* and the non-conserved C-terminal parts of *D. rerio*, *S. pombe* and *S. cerevisiae* have been omitted. In (b), the ADAT3 N-terminal 16 amino acids longer part of *H. sapiens* (longer human transcript considered (NM_138422), the shorter human transcript (NM_001329533) being equivalent in length to the mouse transcript (NM_001100606)) has been omitted. Secondary structure elements (Sec. Struct.), as observed in the mouse ADAT2/ADAT3 structure, are indicated above the alignments. Residues not found in the electron density and missing in the final model are shown in italics. The ADAT3-specific loop removed for crystallization is shown in grey. Residues of ADAT2 and ADAT3 involved in zinc binding are indicated with yellow stars. ADAT2 glutamate potentially involved in proton shuttling is indicated with a blue star. ADAT2 residues potentially involved in wobble adenine recognition are shown with purple diamonds. Residues involved in the ADAT3 V128 (red circled yellow diamond) hydrophobic core are indicated with orange diamonds.

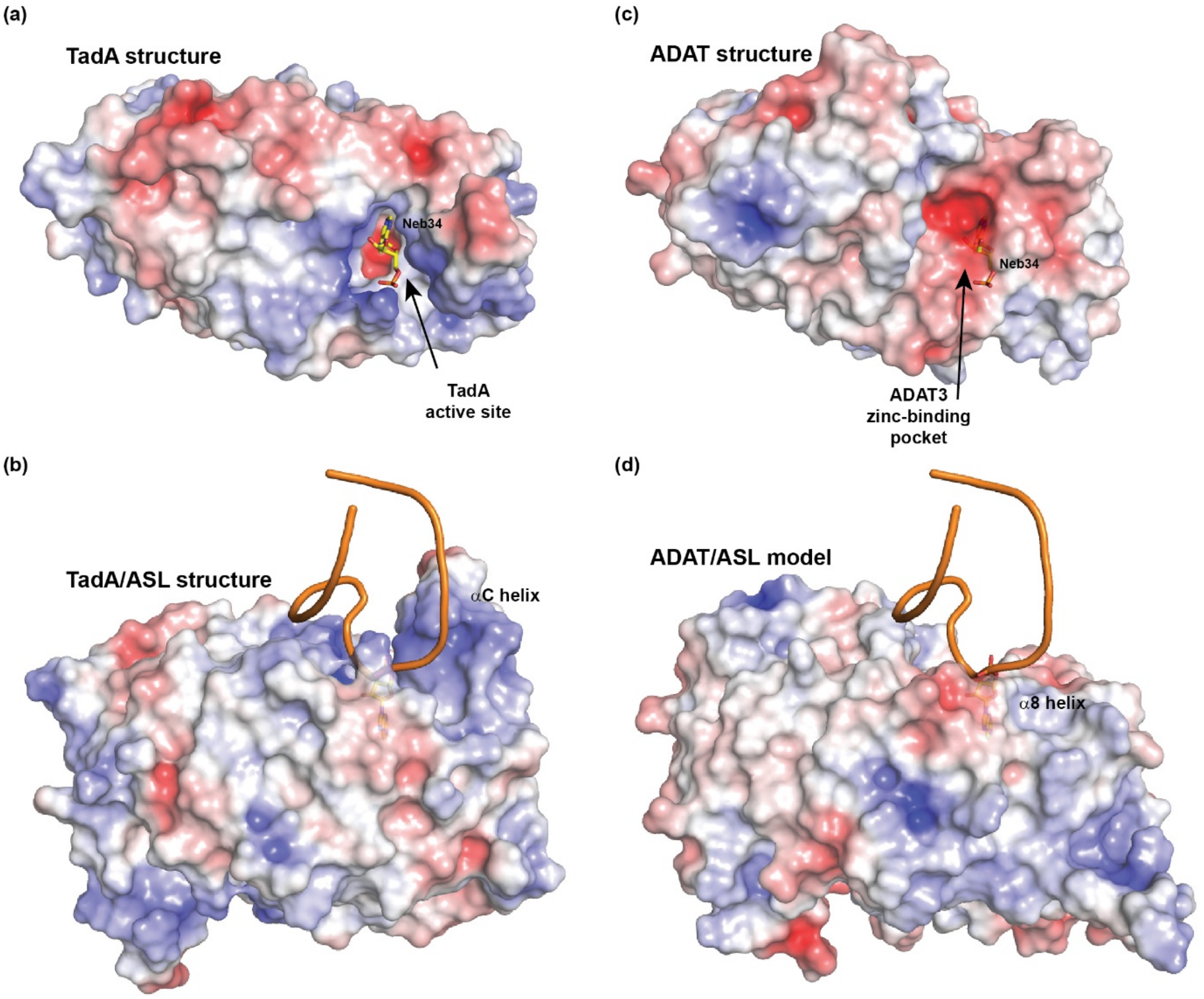

Supplementary Figure 2. ADAT3 active site cannot accommodate an anticodon stem-loop

(a) Binding of a wobble non-hydrolysable adenine analog, nebularine (Neb34), within the active site of *S.aureus* TadA (PDB code 2b3j). The electrostatic potential at the TadA homodimer surface is displayed (blue, positively charged; red, negatively charged). **(b)** Same as in (a) in a rotated view with the ASL shown as orange ribbon. The αC-helix of TadA participates to the formation of the groove that recognizes the ASL. **(c)** Model same as in (a) with the ADAT3 zinc-binding site of the ADAT complex. There is no pocket for the wobble nucleoside due to the capping of the zinc-binding site by ADAT3 C-terminus. In addition, the negative (red) electrostatic character of the ADAT3 surface would be repulsive for binding of an ASL. **(d)** Model same as in (b) with the ADAT complex and the ASL binding to ADAT3 zinc-binding site. The α8 helix of ADAT3 is too short to help form an ASL recognition groove as observed in TadA.

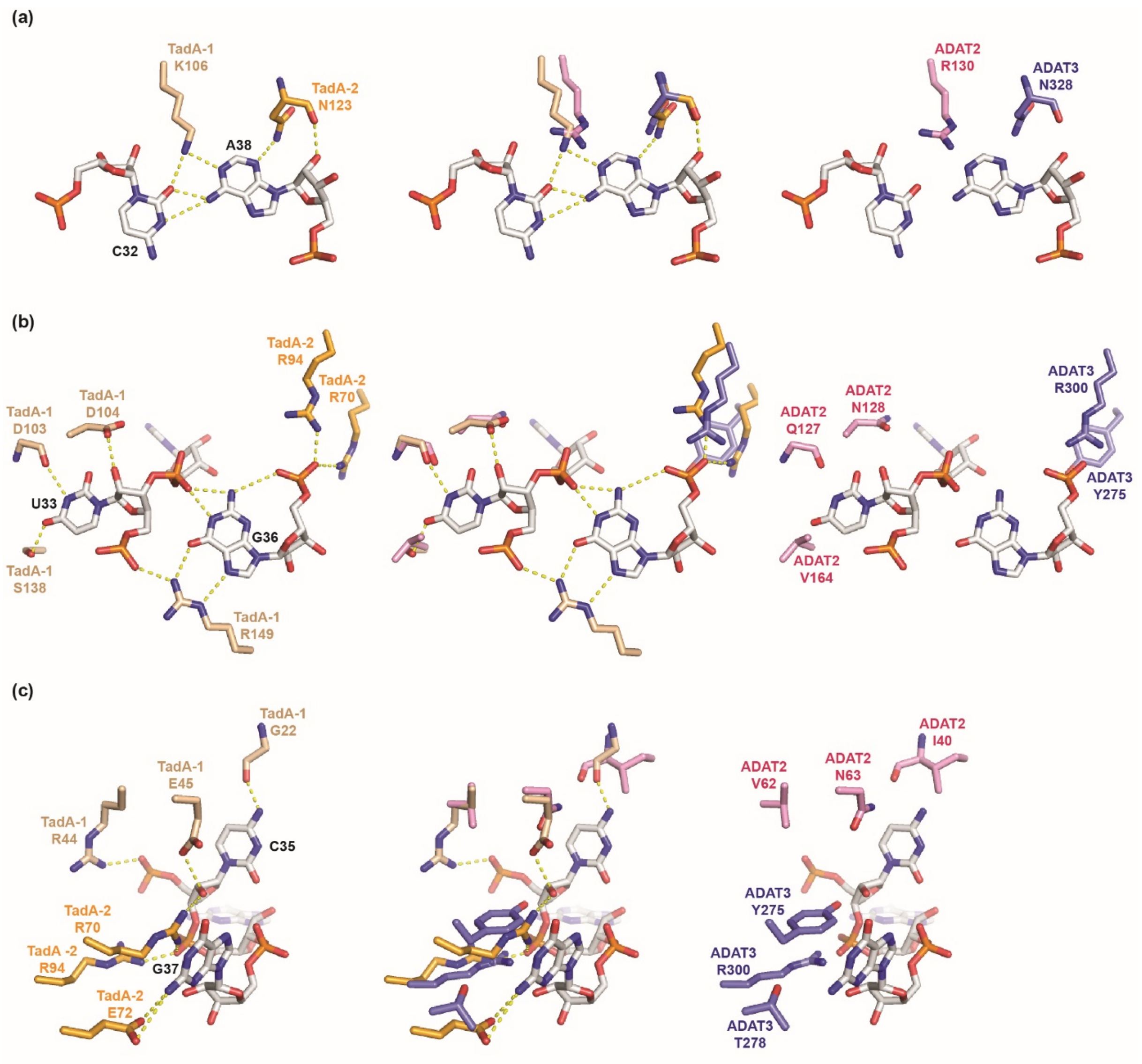

Supplementary Figure 3. ADAT anticodon loop recognition pockets diverge from those of TadA

(a-c) Anticodon loop recognition pockets of TadA active site (left panels) and of ADAT2 active site (right panels) and their superposition (middle panels). Residues involved in interactions are shown as sticks with different colours depending on whether they belong to TadA first monomer (TadA-1, coloured sand) or second monomer (TadA-2, coloured orange), or to ADAT2 (coloured pink) or ADAT3 (coloured blue). Some TadA-equivalent residues are not shown in the ADAT pockets due to their non-existence in the ADAT2 and ADAT3 proteins. While recognition of the 32-38 pair (a) appears conserved between TadA and ADAT, recognition of the 33 and 35 nucleosides has lost some determinants, the strongest loss of determinants concerning nucleosides 36 and 37 (b-c).

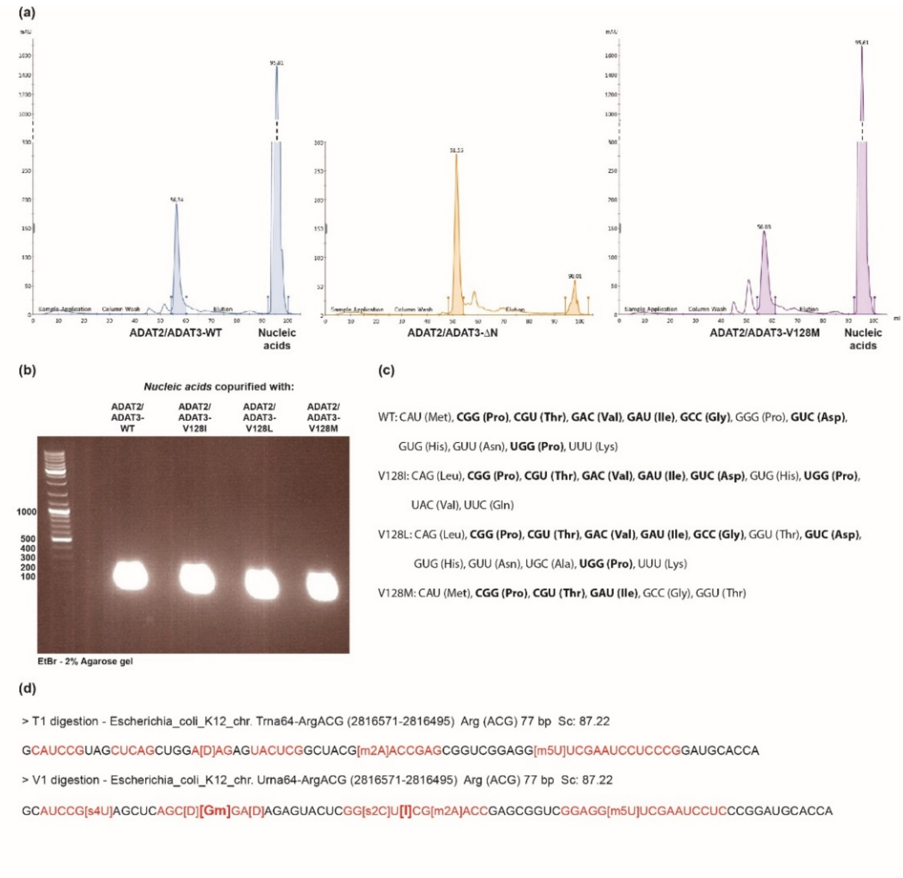

Supplementary Figure 4. **Mouse ADAT binds to various *E. coli* tRNAs and *E. coli* TadA co-purifies with *E. coli* tRNA^{Arg}(ICG) having an unexpected Gm$_{18}$ modification.**

(a) Ion exchange purification profiles for ADAT2/ADAT3 WT (left panel), ADAT2/ADAT3-ΔN (middle panel) and ADAT2/ADAT3-V128M (right panel). The ADAT2/ADAT3-V128I and ADAT2/ADAT3-V128L mutants purification profiles are not displayed but show the same profile as the WT or the V128M mutant. A large peak of nucleic acids is observed for both the WT and the V128M mutant (signal above 1500 mAU), whereas a residual peak (30 fold decrease in height) is observed for the ΔN mutant (signal below 100 mAU), demonstrating that ADAT3 N-terminal region is important for nucleic acid binding. **(b)** Analysis on agarose gel of the nucleic acids co-purifying with the ADAT2/ADAT3 WT, V128I, V128L and V128M mutants. All nucleic acids migrate around the 100 bp marker and have been shown by mass spectrometry analysis to be exclusively a mix of *E. coli* tRNAs. **(c)** Anticodons (and corresponding amino acids) of tRNAs unambiguously characterized in the mass spectrometry analysis. Anticodons found in at least three of the four samples are shown in bold. **(d)** Result of the mass spectrometry sequencing analysis of the *E. coli* tRNA^{Arg}(ACG) co-purifying with *E. coli* TadA after its digestion with RNAses T1 and V1. The red sequences indicate the identified regions. The methylated G$_{18}$ and the wobble inosine identified upon T1 digestion are indicated in bold. The full modification of the wobble adenosine into inosine of this tRNA was also observed by sequencing. Note that some modifications like pseudouridine cannot be detected due to equivalent mass with the non-modified base.

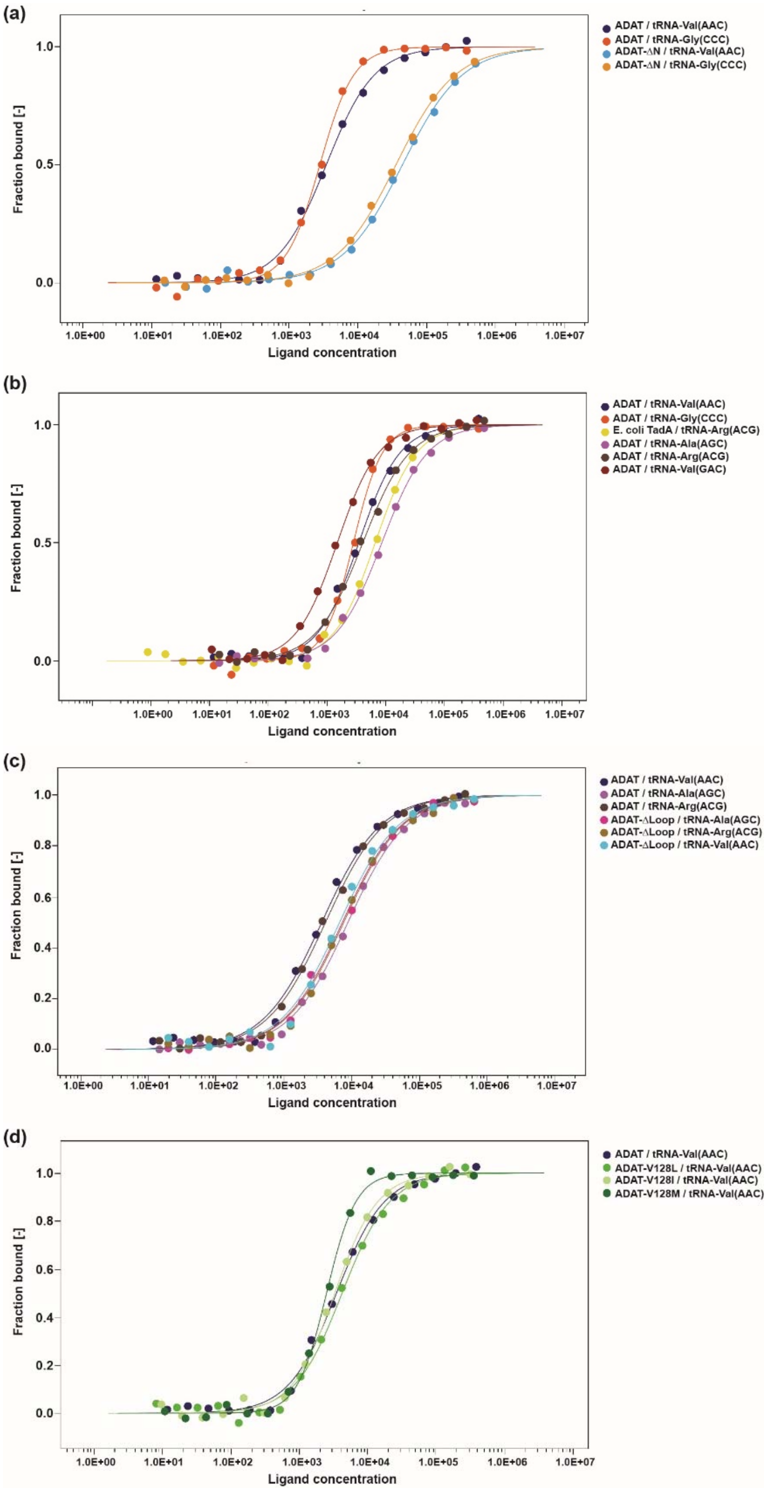

Supplementary Figure 5. **Assessment of tRNAs binding to ADAT and TadA**

(a,b,c,d) Measurements by Microscale Thermophoresis (MST) of the Kd of various tRNAs for mouse ADAT (both WT and mutants) and *E. coli* TadA. The Kd values can vary between the different tRNAs. ADAT can even bind to non-cognate tRNAs. Binding is dependent on ADAT3-specific loop, as assessed by the ΔLoop mutant, and notably on ADAT3 N-terminal domain, as assessed by the ΔN mutant. The disease-causing mutation V128 does not prevent tRNA binding.

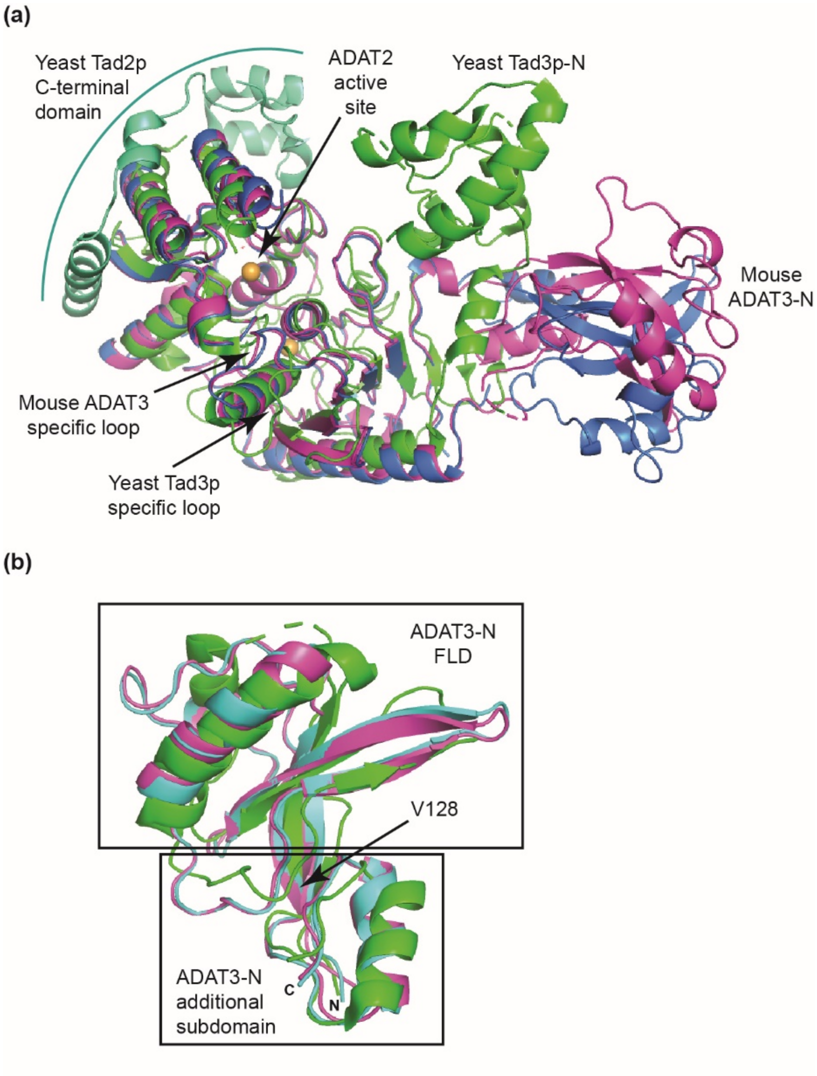

Supplementary Figure 6. **Comparison of mouse and yeast ADAT complexes.**

(a) Superposition of both mouse ADAT2/ADAT3 structures (blue and magenta) and yeast Tad2p/Tad3p structure (coloured green). The mouse and yeast complexes show structural similarities and dissimilarities. Specifically, yeast Tad2p has an additional C-terminal domain (green-cyan), not present in mouse ADAT2, whose function is unknown and that wraps around Tad2p deamination domain. Mouse ADAT3-N and yeast Tad3p-N domains are indicated with a significant difference in positioning, although their movement could bring them in similar positions compared to their respective catalytic domains. The position of mouse and yeast ADAT3 specific loops are indicated. The mouse loop is closer to ADAT2 active site, in agreement with a potential role in tRNA positioning for catalysis, as suggested by our deamination assays. **(b)** Superposition of the ADAT3-N domains of mouse and yeast ADAT complexes displayed from their tRNA binding sides. The colour code is the same as in (a). Despite a similar fold, significant structural differences are observed for both their FLDs and their additional structural subdomains. Specifically, the structural environment of mouse V128 is different in both complexes.

Supplementary Table 1. Anticodon loops recognized by human/mouse ADAT and E. coli TadA

**tRNA**	**Position 32**	**Position 33**	**Position 34 (wobble)**	**Position 35**	**Position 36**	**Position 37**	**Position 38**
Hs/Mm* ALA (AGC)	U	U	**A**	G	C	A	U
Hs/Mm ARG (ACG)	C	U	**A**	C	G	G	A
Hs/Mm ILE (AAU)	C	U	**A**	A	U	A	A
Hs/Mm LEU (AAG)	U	U	**A**	A	G	G	C
Hs/Mm PRO (AGG)	U	U	**A**	G	G	G	U
Hs/Mm SER (AGA)	C	U	**A**	G	A	A	A
Hs/Mm THR (AGU)	C	U	**A**	G	U	A	A
Hs/Mm VAL (AAC)	C	U	**A**	A	C	A	C
Hs/Mm All cognate tRNAs	C/U	U	**A**	A/C/G	A/C/G/U	A/G	A/C/U
Hs/Mm GLY (CCC)	*C/U*	*U*	***C***	*C*	*C*	*A*	*U*
E. coli ARG (AGC)	*C*	*U*	***A***	*C*	*G*	*A*	*A*

*Hs, Homo sapiens; Mm, Mus musculus.

Supplementary Table 2. Crystallographic table.

**Data collection***	**mADAT WT Structure 1**	**mADAT WT Structure 2**	**mADAT V128L**
Space group	P 3_2 2 1	P 2 2_1 2_1	P 2 2_1 2_1
Cell dimensions			
a, b, c (Å)	105.47, 105.47, 187.08	52.29, 106.83, 130.58	51.98, 106.81, 129.67
α, β, γ (°)	90.0, 90.0, 120.0	90.0, 90.0, 90.0	90.0, 90.0, 90.0
Resolution (Å)	50. – 2.96 (3.13 – 2.96)	50. – 2.12 (2.25 – 2.12)	50. – 1.99 (2.11 – 1.99)
Rsym or Rmerge	9.2 (381.9)	18.0 (242.0)	13.6 (244.4)
I / σI	26.48 (1.02)	11.57 (1.05)	15.15 (1.05)
Completeness (%)	99.9 (99.9)	99.8 (99.1)	99.8 (99.1)
Redundancy	19.7 (19.7)	13.3 (13.3)	13.2 (12.4)
CC(1/2) (%)	100.0 (54.5)	99.8 (52.2)	99.9 (38.5)
**Refinement**			
Resolution (Å)	45.94 – 2.96	49.44 – 2.12	49.38 – 1.99
No. reflections	25868	42204	49990
Rwork / Rfree	0.195 / 0.233	0.201 / 0.233	0.189 / 0.218
Number of atoms			
Protein	3446	3437	3423
Ions	2	2	2
Waters	1	167	288
B-factors			
Protein	122.51	49.24	46.47
Ions	102.65	35.35	32.01
Waters	102.69	46.46	49.34
R.m.s. deviations			
Bond lengths (Å)	0.009	0.008	0.007
Bond angles (º)	1.065	0.880	0.857
** Values in parentheses are for the highest-resolution shells.*			

Supplementary Table 3. Deamination assays.

	tRNA-Val(AAC)			tRNA-Arg(ACG)			tRNA-Ala(AGC)			tRNA-Gly(ACC)		
	1:3[a]	*1:0.3*	*1:0.03*	*1:3*	*1:0.3*	*1:0.03*	*1:3*	*1:0.3*	*1:0.03*	*1:3*	*1:0.3*	*1:0.03*
**ADAT2/ADAT3**	98.0±1.4[b]	75.5±6.4	5.5±0.7	83.0±1.4	65.5±12.0	7.5±10.6	84.0±1.4	73.0±0	69.0±4.2	18.0±1.4	0	0
**ADAT2/ADAT2**	1.0±1.1	*n.d.*[c]	*n.d.*	0	*n.d.*	*n.d.*	7.5±0.7	*n.d.*	*n.d.*	*n.d.*	*n.d.*	*n.d.*
**ADAT3**	0	*n.d.*	*n.d.*	0	*n.d.*	*n.d.*	0	*n.d.*	*n.d.*	*n.d.*	*n.d.*	*n.d.*
**TadA/TadA**	0	*n.d.*	*n.d.*	94.5±4.9	36.0±9.9	0	0	*n.d.*	*n.d.*	*n.d.*	*n.d.*	*n.d.*
**ADAT2/ADAT3-ΔNter**	0	*n.d.*	*n.d.*	3.5±2.1	*n.d.*	*n.d.*	0	*n.d.*	*n.d.*	*n.d.*	*n.d.*	*n.d.*
**ADAT2/ADAT3-Acidic1**	15.0±8.5	0	0	12.0±0	1.0±1.1	1.0±1.1	74.5±0.7	68.5±3.5	15.5±21.9	*n.d.*	*n.d.*	*n.d.*
**ADAT2/ADAT3-Acidic2**	0	0	0	1.0±0	0	0	0	0	0	*n.d.*	*n.d.*	*n.d.*
**ADAT2-E73A/ADAT3**	0	*n.d.*	*n.d.*	2.0±1.4	*n.d.*	*n.d.*	11.0±0	3	0	*n.d.*	*n.d.*	*n.d.*
**ADAT2-E73A/ADAT3-V225E**	0.5±0.7	*n.d.*	*n.d.*	0	*n.d.*	*n.d.*	0	*n.d.*	*n.d.*	*n.d.*	*n.d.*	*n.d.*
**ADAT2-E73A/ADAT3-ΔCter**	2.0±2.8	*n.d.*	*n.d.*	2.5±3.5	*n.d.*	*n.d.*	64.0±1.4	0	0	*n.d.*	*n.d.*	*n.d.*
**ADAT2/ADAT3-Δloop**	15.0±0	3.5±2.1	0	44.0±1.4	5.0±4.2	0	71.5±0.7	69.0±2.8	3.5±4.9	*n.d.*	*n.d.*	*n.d.*
**ADAT2/ADAT3-V128M**	76.0±12.7	32.0±5.7	0	80.5±0.7	8.5±7.8	0	80.5±0.7	70.5±0.7	41.0±31.1	*n.d.*	*n.d.*	*n.d.*

[a]tRNA/enzyme ratio

[b]mean value ± standard deviation from duplicates

[c]n.d., not determined. For most samples, the dilution 1:0.3 and 1:0.03 were only tested once and not tested again if (i) no deamination was observed for these dilutions in the first measurement and (ii) the 1:3 dilution first measurement was close or equal to 0. These single measurements have been marked as not determined. The $tRNA^{Gly}$(ACC) was only tested with WT ADAT.
